# A network approach to zooarchaeological datasets and human-centered ecosystems in southwestern Florida

**DOI:** 10.1371/journal.pone.0295906

**Published:** 2023-12-19

**Authors:** Isabelle Holland-Lulewicz, Jacob Holland-Lulewicz

**Affiliations:** Department of Anthropology, The Pennsylvania State University, University Park, Pennsylvania, United States of America; New York State Museum, UNITED STATES

## Abstract

Zooarchaeological datasets are often large, complex, and difficult to visualize and communicate. Many visual aids and summaries often limit the patterns that can be identified and mask interpretations of relationships between contexts, species, and environmental information. The most commonly used of these often include bar charts, pie charts, and other such graphs that aid in categorizing data and highlighting the differences or similarities between categories. While such simplification is often necessary for effective communication, it can also obscure the full range of complexity of zooarchaeological datasets and the human-environment dynamics they reflect. In this paper, we demonstrate the utility of formal network graphs to capturing the complexity of zooarchaeological datasets and to effectively highlighting the kinds of relationships between contexts, time, and faunal assemblages in which zooarchaeologists are primarily interested. Using a case study from southwestern Florida (USA), we argue that network graphs provide a quick solution to visualizing the structure of zooarchaeological datasets and serve as a useful aid in interpreting patterns that represent fundamental reflections of human-centered ecosystems.

## Introduction

The zooarchaeological record is complex, cumbersome, and often difficult to effectively conceptualize, communicate, and visualize. While simplification is often necessary for effective communication, it also obscures the range of complexity inherent in these datasets. In this paper, using a case study from southwestern Florida, USA, we demonstrate the utility of formal network graphs to capturing the complexity of zooarchaeological datasets and to highlighting relationships between contexts, time, and taxa. At its core, a network approach can be a powerful tool for visualizing and interpreting zooarchaeological data, and more broadly for illuminating fundamental reflections of past human-centered ecosystems.

Here, we aim to visualize, track, and analyze past human-centered ecosystems reflected in the zooarchaeological remains of a large, precontact town in southwestern Florida. The dense, extensive deposits of marine fauna across this region make it well suited to exploring long-term human-ecosystem dynamics, in this case relationships between humans, marine species, time, and space across the first millennium AD. The ecological data we leverage are the discarded animal remains, especially invertebrates, or shellfish, that were either intentionally caught and exploited by humans, captured and discarded as unintentional by-catch, and those species that colonized shoreline trash heaps created by humans. The composition of these assemblages is fundamentally driven by, and centered around, human action and histories. The networks we present are fundamental reflections of these human-centered ecosystems.

The Pineland Site Complex (8LL33, etc.), a Calusa site in southwestern Florida, harbors an approximately 1700-year occupational history of long-term socioecological relationships, or rather human-centered ecosystems. Researchers at the Florida Museum of Natural History conducted an over 30-year long research program aimed at, among other things, elucidating this relationship between cultural and natural processes [1]. The landscape of southwestern Florida is extremely dynamic and houses a rich and diverse set of resources extensively utilized by Indigenous people as demonstrated by extensive zooarchaeological research on contexts not only at the Pineland Site but also from several others located within the same estuarine system. In fact, the Pineland Site possesses the most extensive zooarchaeological assemblages from a single site in southwestern Florida, a product of such a long-term research program.

Previous zooarchaeological research at the Pineland Site was centered on examining the linkages between paleoenvironmental data, i.e. the zooarchaeological data, and the occupational history of the site as well as changes in subsistence practices at different temporal and spatial scales [1, 2]. More specifically, this previous study uses the totality of zooarchaeological data generated from Pineland as a composite data set representing the site as a whole with which to compare more specific contexts and temporal trends within the site itself, to examine different depositional environments including waterlogged and storm deposits, and to examine trends in paleoenvironmental conditions. Given the diversity and sensitivity of these ecosystems and the extensive amount of zooarchaeological data generated, network graphs provide a way to quickly visualize the structure of these data sets and provide a useful tool to interpreting patterns in the transformation of these human-centered ecosystems beyond the traditional methods employed in this previous study. More specifically, the study presented here allows for a more bottom-up, inductive approach to zooarchaeological data. In this way, our analyses highlight important variation and deviations from previous results structured around top-down approaches that begin with rote categorization as the basis for analyses (e.g., domestic v. non-domestic, mound v. non-mound, time periods I-IV, etc.). Indeed, from a network perspective, we highlight temporal and spatial heterogeneity in the scale and structure of the human-centered ecosystems reflected in the archaeological record of Pineland.

### Network analysis and socioecologies

Across ecologically focused disciplines, network analyses have been leveraged as Ecological Network Analysis (ENA). In the last decade or so, the major themes informed by this work have been food web ecosystems, species loss, flow and sustainability, security, systems ecology and ecosystems, risk assessment, energy and urban metabolism, and wetland water systems, with work on food webs, energy and metabolism, and systems ecology representing most efforts [3]. Overall, the broad goal of ENA applications is to investigate ecosystem structure and function [3] and is understood to be an effective ecosystems-based approach to resource management [4]. Not unlike other applications of network analysis, indeed in many ways theoretically and methodologically analogous to its use in the social sciences, ENA is a “systems-oriented methodology to analyze within system interactions to identify holistic properties that are otherwise not evident from direct observations” [5]. The majority of applications of ENA have been directed towards understanding trophic relationships in the context of food webs and broader ecosystem functioning. In this regard, ENA has offered a powerful toolkit for visualizing and assessing the complexity of relationships, both direct and indirect, between different taxa, taxa groups, and both biotic and abiotic components of an ecosystem [6]. Importantly, ENA has provided ecologists with a tool for approaching challenges and analyses from the level of the entire ecosystem, inclusive of component parts, and the complex relationships between those parts that give form to the holistic system.

To address modern challenges described as “cross-sectoral, multi-level, and trans-boundary,” such as issues of watershed restoration, species conservation, or fisheries management, Socio-Ecological Network Analysis (SEN) has emerged as a way to formally evaluate complex human-environment dynamics [7–10]. An SEN, by definition, addresses both social and ecological phenomena, their interactions, and social and ecological processes, concepts, and theories must recursively underpin the study [7]. For example, work by Crabtree et al. [11] reconstructed human-centered food webs in the western desert of Australia to understand how the role and position of humans in broader ecological systems shifted with the impacts of European colonization and the movement from exclusively foraging-based lifeways to mixed-based economy lifestyles. In this study, all species within the ecosystem were included as nodes and ties between them, representing paths of consumption, and were analyzed to understand both the structure of a complex socioecological system as well as how adding, removing, or altering relationships between nodes affected that structure.

While archaeologists have likewise adopted social network analyses (SNA) [12–16], most studies have focused on reconstructing and analyzing past social networks such as political networks, geographic networks, and other kinds of interaction networks the defined past societies. The archaeological data that most often underpin archaeological network analyses include ceramic technologies and decorations [17–20], geochemical, raw material, or geomorphometric information on lithic sources [21–25], or other kinds of prestige goods or crafts [26–28].

To date, few archaeological studies have explicitly leveraged network analyses to explore past, or long term, socio-ecologies, nor have such analyses been readily applied to the kinds of ecological data that archaeological sites preserve [29]. As zooarchaeological, and even botanical, data could certainly reflect past interactions both among people and between people and ecologies, there is a valuable opportunity to explore the application of these methods to such datasets. Indeed, as demonstrated through its use across ecological and socioecological cases, network analyses are well suited to the ecological datasets yielded through archaeological research. It has the potential to contribute substantively to the study of past human-centered ecosystems and may serve as a powerful tool in what Crabtree and Dunne [30] call the science of “archaeocology,” the examination of the past 60,000 years of interactions between humans and ecosystems.

Crabtree et al. [30] provide a series of example questions that socioecological network analysis could be used to address using archaeological data. These include inquiries such as: how do the effects of hunting and gathering by humans propagate through an ecosystem?; how do humans positively and negatively affect ecosystem diversity?; how do humans respond to and potentially mediate effects of the physical environment?; and how to species colonization or extinctions affect ecosystems? One of the most explicit examples of such work comes from Verhagen et al. [31] who leveraged network analyses and archaeological data on plant and animal species to reconstruct “human-centered interaction networks” during the Neolithic transition in the Netherlands. The purpose of these reconstructions was to better understand the role of culture, ecology, and environment in the long-term evolution of a particular socio-ecological system. As these archaeoecological data are the result of direct and indirect interactions with humans, they are fundamental representations of human-centered ecosystems, and as such, the changes to these ecosystems through time and space can be tracked.

In this context, the case study presented here for southwestern Florida seeks to address questions related to human-ecological dynamics over the course of a millennia. More specifically, we ask: what kinds of ecological networks were the Calusa embedded in along the southwestern Gulf Coast of Florida? How were different parts of this estuarine and marine ecosystem leveraged at different times? How did different parts of this ecosystem manifest variably across the Calusa town of Pineland? What was the role or place of humans in long-term ecosystem change? How did these long-term changes, driven by climate change, alter the character of human-center ecosystems at Pineland?

### Socioecological histories of southwestern Florida

**Fig 1:**
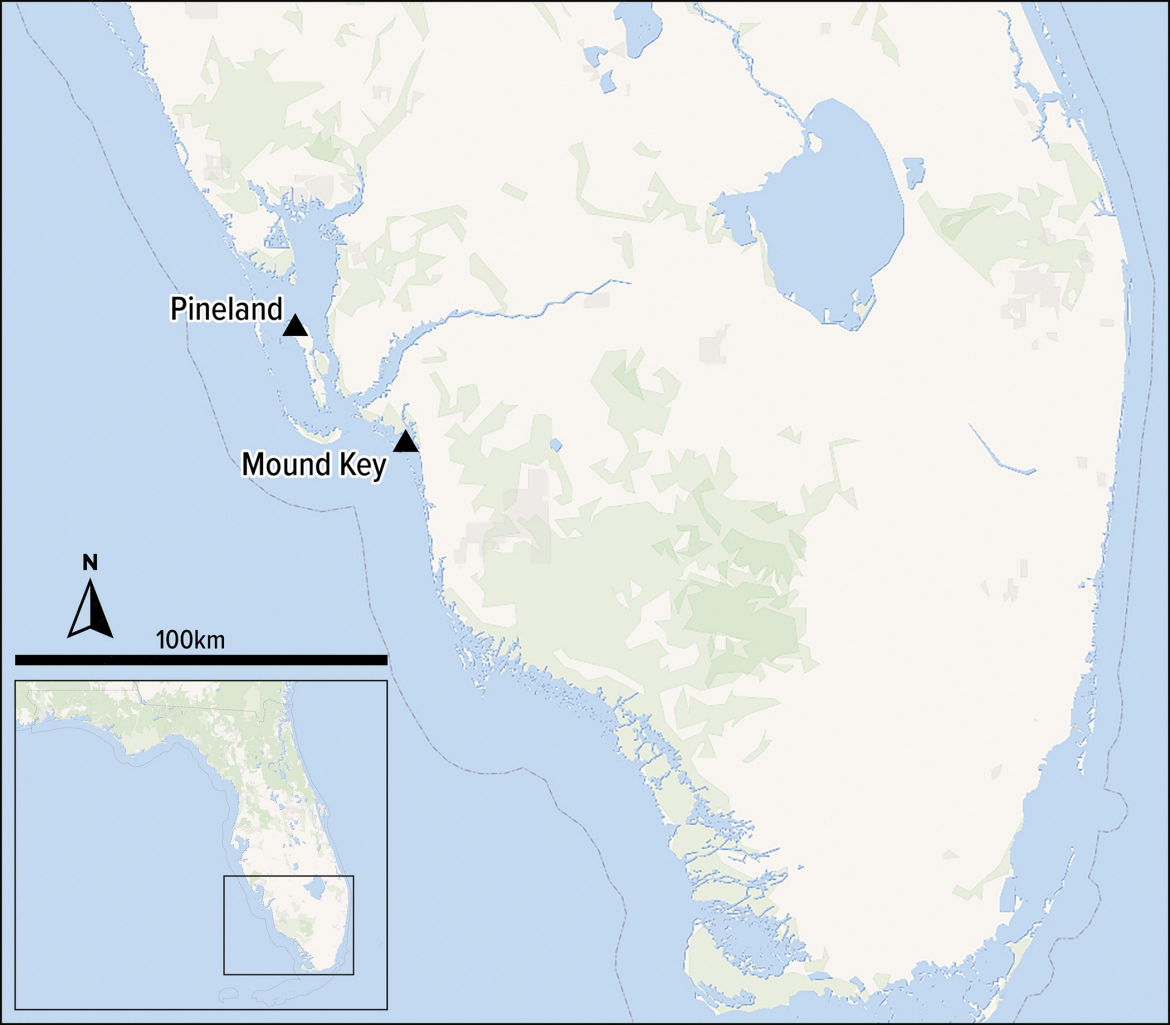
Southwestern Florida indicating locations of Pineland Site Complex (8LL33, etc.) and Mound Key (8LL2). Source: *OpenStreetMap contributors*. *OpenStreetMap database [PostgreSQL via API]*. *OpenStreetMap Foundation*: *Cambridge*, *UK; 2023 [cited Sep 2023]*. *© OpenStreetMap contributors*. *Available under the Open Database Licence from*: *openstreetmap*.*org*. *Data mining by OpenMapTiles*, *openmaptiles*.*org*.

The Calusa were a non-agricultural, complex chiefdom or weak tributary state, which exacted control over the lower third of peninsular Florida at the time of European contact in the sixteenth century (Fig 1). In the absence of maize, the Calusa relied on aquatic resources, wild plant foods, and cultivated home gardens providing plants like chili peppers, papaya, gourds, and squash. Environmental conditions undoubtedly played a key role in both sociopolitical and socioeconomic structuring and restructuring of Calusa communities given their reliance on fishing, gathering, and hunting. Over a roughly 800-year period of fluorescence, Calusa lived in dense urban settlements, developed complex institutions to manage fish resources, and profoundly terraformed the landscape. The Calusa capital, Mound Key, is a 65-hectare anthropogenic island made entirely of discarded shell and is characterized by monumental architecture such as mounds, canals, and watercourts for live fish surplus [32, 33]. Here we focus on the Calusa town of Pineland, located approximately 40 kilometers north of Mound Key on Pine Island (Fig 1).

**Fig 2:**
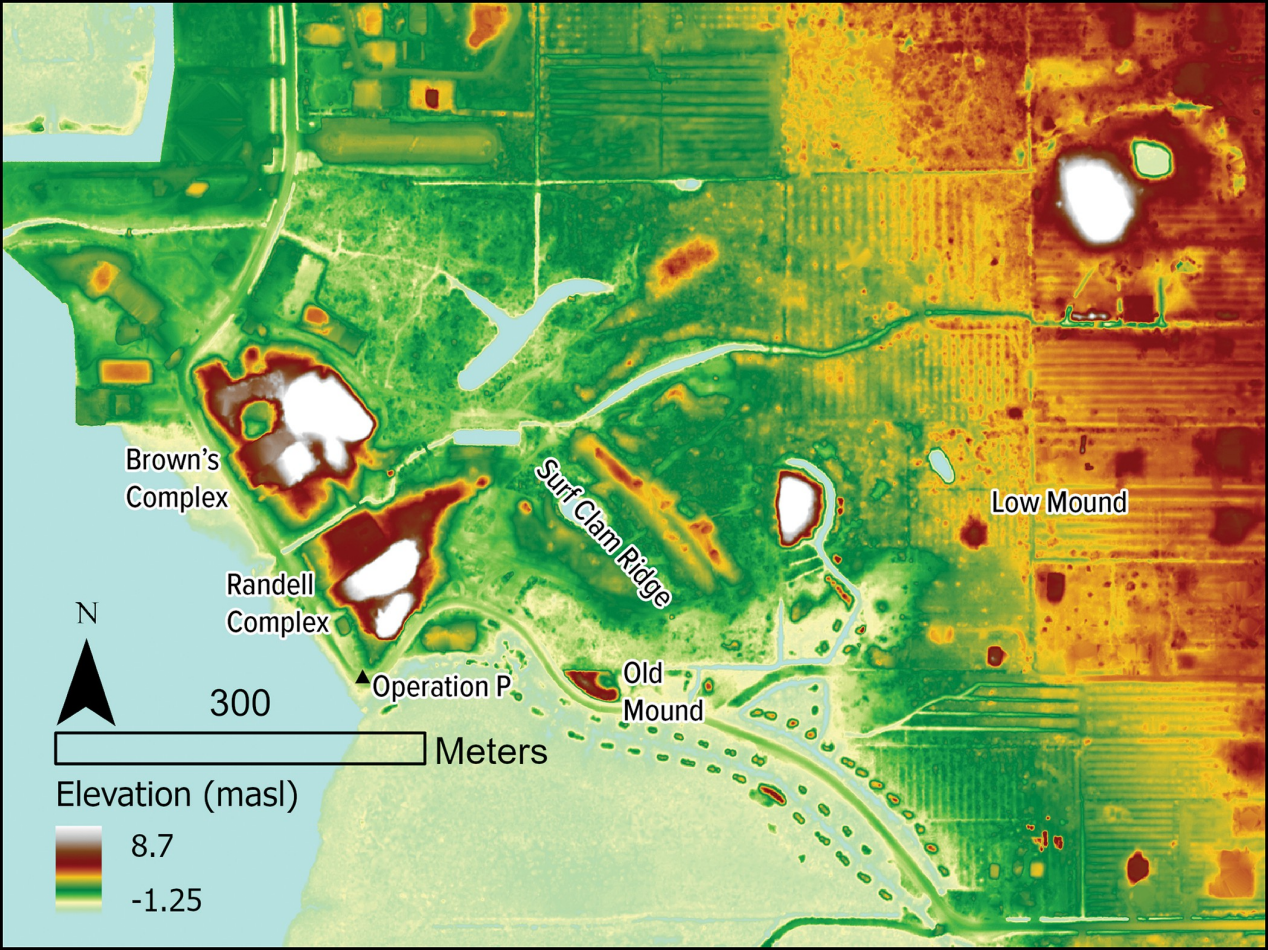
Pineland Site Complex indicating locations from which zooarchaeological assemblages used here have been analyzed. Data Source: NOAA Data Access Viewer, coast.noaa.gov/dataviewer.

Extensive work across the Pineland Site Complex (8LL33, etc.) (Fig 2), and southwestern Florida more broadly, has yielded a cultural chronology referencing major sociopolitical changes roughly correlated with critical trends in climatic and environmental histories [1, 2, 34, 35] (Table 1). Previous investigations have laid extensive groundwork elucidating transformations to socioecological histories at the Pineland site and other closely related sites within this same estuary [1, 36–38]. The largest of these, the Pineland Site Complex, is a group of related sites, harboring a wide variety of archaeological features resultant from an approximately 1700-year span of occupation established through ceramic chronological placement and a series of radiocarbon dates [1]. Two prominent midden-mound complexes, Brown’s Complex and Randell Complex, are situated on the western portion of the site and are bisected by the 4km long Pine Island Canal (8LL34) [1, 39, 40]. Numerous other features from throughout the site’s Indigenous occupation are located along this canal including several water courts or ponds, other canals, and numerous other ridges and mounds.

**Table 1:** Generalized chronology of southwestern Florida social, political, and climatic histories.

DATES	Greater North Atlantic Climate	Southwestern Florida Sea Level	Caloosahatchee Cultural Periods	Pineland Chronology (Generalized from Holland-Lulewicz and Thompson 2021; Marquart and Walker 2013:803, Fig 5)						
AD 1800	Little Ice Age	Sanibel II Low	Seminole							
AD 1750										
AD 1700			Caloosahatchee V							
AD 1650										
AD 1600										
AD 1550								Brown’s Complex		Operation P Excavations, Randell Complex Midden
AD 1500										
AD 1450			Caloosahatchee IV							
AD 1400										
AD 1350										
AD 1300			Caloosahatchee III							
AD 1250									Randell Complex	
AD 1200										
AD 1150	Medieval Warm Period	La Costa High	Caloosahatchee IIB							
AD 1100										
AD 1050										
AD 1000										
AD 950										
AD 900										
AD 850										
AD 800	Vandal Minimum	Buck Key Low								
AD 750			Caloosahatchee IIA							
AD 700										
AD 650							Old Mound			
AD 600				Surf Clam Ridge						
AD 550			Caloosahatchee I							
AD 500										
AD 450	Roman Warm Period	Wulfert High								
AD 400					Low Mound	Citrus Ridge				
AD 350										
AD 300										
AD 250										
AD 200										
AD 150										
AD 100										
AD 50										
50 BC										
100 BC										

We note and acknowledge here that many of these dates establishing the chronology for the Pineland Site and vis-à-vis the socioenvironmental histories are acquired from various species of marine shell. Prior to this paper, these dates were not reevaluated with the newly established Marine 20 radiocarbon calibration curve and updated reservoir correction values. A reevaluation of these dates with the new curve, based on how significantly it was updated in 2020, could shift the chronological sequence of the Pineland Site, since many of these dates are on marine shell. However, for the purpose of this paper, we use the previously established, currently accepted chronology to showcase this particular methodology.

During the Caloosahatchee I-late period (AD 1–500), the southwestern portion of the site saw domestic occupations along a proposed old shoreline including named features such as Old Mound (8LL37), Surf Clam Ridge, Citrus Ridge, and Low Mound (8LL1612) with likely year-round occupation [1]. Domestic structures were likely small, round structures (ca. 3.5m in diameter) [41, 42]. The Caloosahatchee IIA period (AD 500–800) contains evidence for settlement shifting to the areas of Old Mound and Brown’s Complex aligning with a global climatic shift known as the Vandal Minimum (ca. AD 550–850). During this climatic interval, conditions were generally cool and dry but with erratic changes to temperature, precipitation patterns, and relative sea-level [43–45]. People likely occupied Pineland year-round yet potentially shifted household organization from small family units to large multifamily households [1, 41]. Marquardt and Walker [1] also posit that during this time fisheries productivity diminished and evidence for long-distance exchange increased.

At the onset of the Medieval Warm Period and the beginning of the Caloosahatchee IIB period (AD 800–1200), the town’s structure shifted from a pattern of paralleling the shoreline to investment in mound architecture situated perpendicular to the shoreline [1]. It is also likely during this time that, as relative sea levels rose, additional water-control features such as lakes, watercourts, other water impoundment features, and canals, including the Pine Island Canal (8LL34), were constructed [40]. The Caloosahatchee III and IV periods (ca. AD 1200–1350 and AD 1350–1500 respectively), coeval with the Little Ice Age (ca. 1250–1800), characterized Pineland as a time where accumulation continued throughout the site especially in association with the mound complexes, people maintained all the water-impoundment features, and across the region people coalesced into larger villages as increasing socio-political complexity accelerated in the region [46]. Intact deposits based on radiocarbon dating and ceramics that post-date this period, within the Caloosahatchee V (AD 1500–1700), have not been identified at Pineland, but isolated objects such as glass beads and other European trade goods have been recovered [1, 47].

Throughout the entirety of Pineland’s occupation between c. AD 150 and 1800, a number of global warming and cooling events manifest locally mentioned briefly above, albeit the degree to which this affected local environments in this region is largely unknown. Environmental conditions likely played a key role in the transformations of Calusa sociopolitical and socioeconomic institutional structures over time given their reliance on fishing, hunting, and gathering. For example, Marquardt and Walker [1] propose that an erratic sea-level regression associated with the Vandal Minimum global climatic episode ca. AD 550–850 created conditions that reduced the availability of certain staple fish. Thompson and colleagues [41] propose a potential restructuring of household organization and thus kin relations and labor roles also sometime during the beginning of this climatic episode. More work is needed to understand the temporality of and relationship between both the local environmental conditions within this particular estuary during this time period and the potentially associated sociocultural shifts. The assemblages used in this analysis and described below are spread across time and space at Pineland and reflect both the shifting ecological and climatic contexts of the Pineland community as well as transformations to the social, political, and economic institutions that formed Calusa society.

### Zooarchaeological assemblages

Through extensive archaeological and zooarchaeological research at Pineland and at sites across the bay, Susan deFrance and Karen Walker have demonstrated Calusa middens in the Charlotte Harbor/Pine Island Sound area are dominated by resources likely collected in immediate proximity to communities [2, 34]. These results suggest zooarchaeological assemblages of both finfish and invertebrates from sites in this region can be used to model local paleoecologies. Fisheries, both invertebrate and vertebrate, are sensitive enough to reflect change at the annual, decadal, and multi-centennial scale making this region a prime locale to explore potential impacts of climatic episodes on local resource regimes. Fundamentally, these assemblages reflect the shifting compositions, content, and connections of human-centered ecosystems through time at Pineland.

In this paper, we collate previously analyzed and published vertebrate and invertebrate data from deFrance and Walker [2] and Holland-Lulewicz and Thompson [36] as this provides an extensive sitewide zooarchaeological dataset (see Table 2). The act of collating zooarchaeological data can be a tricky one depending on screen size, sample size, comparative collection, and analyst skill among many others. Here, we use the zooarchaeological measure of Minimum Number of Individuals (MNI) and percent MNI as a metric for comparison to highlight the number of individuals present and their relative abundance in the various contexts and to avoid disproportionate abundances associated with Number of Identified Specimens (NISP) related to taphonomic processes. All zooarchaeological samples from deFrance and Walker [2] were screened to 1/16^th^ inch (1.6 mm). All invertebrate samples from Holland-Lulewicz and Thompson [36] were screened to 1/16^th^ (1.6 mm) inch while the vertebrate materials were screened only to 1/8^th^ inch (3 mm) due to time constraints. This discrepancy in screen size for these samples likely does not significantly impact any interpretations made in this paper but could underrepresent the some of the smallest of fish captured such as pinfish (*Lagodon rhomboides*), small or juvenile hard head catfish (*Arius felis*), and pigfish (*Orthopristis chrysoptera*) for example (see Quitmyer [48] for small fish species recovered in 1/16^th^ and 1/32^nd^ in screen). Scientific and common names used in this study follow the original identification reporting. Where there were discrepancies between the two datasets in naming convention in genus or species, both were included in Table 2 separated by a forward slash. For more specific zooarchaeological methods, please refer to original data source publications.

**Table 2:** Species list for all Pineland (8LL33, etc.) contexts evaluated. Data are compiled from deFrance and Walker [2] and Holland-Lulewicz and Thompson [36].

		Old Mound 1	Surf Clam Ridge	Low Mound	Old Mound 2	Brown’s Complex 1	Brown’s Complex 2	Brown’s Complex 3	Randell Complex 1	Randell Complex 2	Brown’s Complex Mound 2a	Brown’s Complex Mound 2b	Operation P	Operation P	Operation P	Operation P
Taxon	Common	A-8-101	Trench 11B-93	A-1-77-1	A-16-92	C-6-92-1	C-5-88-2	C-5-79-1	A-Prof-63	A-Prof-55	I-2-73	I-2-66	P-10-99	P-10-101	P-1-103	P-10-105
*Calliostoma* sp.	Topsnail	—	—	—	—	—	—	—	—	—	—	—	—	—	—	1
*Diodora cayensis*	Cayenne keyhole limpet	1	—	—	—	—	—	—	—	—	—	—	—	—	1	3
*Assiminea succinea*	Atlantic assiminea	—	—	—	—	—	—	—	—	—	—	—	13	—	—	—
*Littoridinops* sp.	Hydrobe	—	—	—	—	—	—	—	—	—	—	—	—	—	2	—
*Heleobops docima*	Oolite hydrobe	1	—	—	—	—	—	—	—	—	—	—	—	—	—	—
*Tryonia aequicostata*	Smooth-rib hydrobe	—	—	—	—	—	—	—	—	—	—	—	—	—	1	—
*Littorina angulifera*	Mangrove periwinkle	—	—	—	—	—	—	2	—	—	—	—	—	1	—	—
*Littorina* sp.	Periwinkle	—	—	—	—	—	—	—	—	—	—	—	—	—	—	2
*Modulus modulus*	Buttonsnail	7	—	2	4	4	—	2	4	6	—	5	11	3	35	102
*Cerithidea costata*	Costate hornsnail	1	—	22	—	—	—	—	—	—	—	—	—	—	14	40
*Cerithidea scalariformis*	Ladder hornsnail	533	—	—	—	28	—	22	3	2	—	—	24	5	8	44
*Cerithidea* sp.	Hornsnail	—	—	—	1	—	—	—	—	—	—	—	—	—	—	—
*Schwartziella catesbyana*	Catesby’s Risso	—	—	—	—	—	—	—	—	—	—	—	1	—	—	—
*Truncatella caribaensis*	Caribbean truncatella	6	—	—	—	—	—	—	—	—	—	—	—	—	—	—
*Truncatella pulchella*	Beautiful truncatella	10	—	—	—	1	—	—	—	—	—	—	—	—	—	—
*Turritella exoleta*	Eastern turretsnail	—	—	—	—	—	—	—	2	—	—	—	1	—	—	—
*Turitella variegata*	Variegated turretshell	—	—	—	—	1	—	—	—	—	—	—	—	—	—	—
*Turritella* sp.	Turretsnail	—	—	—	—	—	—	—	1	—	—	—	—	—	—	—
Turritellidae	Turretsnail	2	—	—	—	—	—	—	—	—	—	—	—	—	2	6
Vermetidae	Wormsnails	2	—	1	—	—	—	—	1	1	—	1	—	—	—	1
*Bittium varium*	Grass cerith	—	—	—	—	—	—	—	4	—	—	—	2	—	—	—
*Bittium* sp.	Cerith	—	—	—	—	—	—	—	1	—	—	—	—	—	—	—
*Cerithium algicola*	Middle-spined cerith	1	—	9	—	9	—	—	—	—	—	14	—	—	—	—
*Cerithium eburneum*	Ivory cerith	—	—	—	—	1	—	2	—	1	—	—	2	—	3	3
*Cerithium litteratum*	Stocky cerith	—	—	—	—	—	—	5	—	12	—	26	—	—	—	—
*Cerithium lutosum*	Variable cerith	41	1	21	—	1	—	—	—	—	—	—	80	—	16	15
*Cerithium muscarum*	Flyspeck cerith	53	—	—	—	—	—	378	29	10	1	—	137	92	190	4067
*Cerithium* spp.	Cerith	12	—	—	19	5	2	5	2	32	—	—	—	—	—	—
Cerithiidae	Ceriths	—	—	1	—	—	—	—	—	—	1	—	—	—	—	—
*Seila adamsi*	Adam’s miniature cerith	—	—	—	—	—	—	—	2	—	—	—	1	—	4	2
Cerithopsidae	Common miniature cerith	—	—	—	—	—	—	—	—	—	—	—	—	—	2	1
*Triphora* sp.	Triphora	—	—	—	—	—	—	—	—	1	—	—	—	—	—	—
Euliumidae	Euliumid	—	—	—	—	—	—	—	—	—	—	—	—	—	—	2
*Strombus alatus*	Florida fighting conch	—	—	—	—	—	—	—	5	—	4	—	—	—	13	8
*Crepidula aculeata*	Spiny slippersnail	—	—	—	—	—	—	—	—	5	—	—	—	1	1	—
*Crepidula fornicata*	Common slippersnail	—	—	—	—	1	—	—	—	—	—	21	10	2	33	—
*Crepidula maculosa*	Spotted slippersnail	—	—	—	—	—	—	—	—	—	—	—	—	—	16	—
*Crepidula plana*	Eastern white slippersnail	12	—	31	5	—	—	17	54	14	—	13	4	6	19	—
*Crepidula* spp.	Slippersnail	51	—	28	2	13	—	8	82	15	2	—	—	—	—	74
*Cerodrillia thea*	Tea drillia	—	—	—	—	—	—	—	—	—	—	—	1	—	—	2
*Cerodrillia (cf*.*) thea*	Tea drilla	—	—	—	—	—	—	—	—	—	—	—	—	—	1	—
*Neverita duplicata*	Shark eye	1	—	5	19	1	—	25	5	5	4	2	2	2	3	—
*Sinum perspectivum*	White baby-ear	—	—	1	—	—	—	—	—	—	—	—	—	—	—	—
*Eupleura sulcidentata*	Sharp-rib drill	—	—	—	—	—	—	—	—	—	—	—	—	2	2	8
*Urosalpinx cinerea*	Atlantic oyster drill	—	—	—	3	1	—	2	7	8	—	—	4	2	5	25
*Urosalpinx perrugata*	Gulf oyster drill	2	—	—	—	—	—	—	—	—	2	—	6	4	9	25
*Urosalpinx tampanensis*	Tampa drill	—	—	—	—	—	—	—	—	—	—	8	—	—	1	7
*Anachis pulchella*	beautiful dovesnail	—	—	—	—	5	—	—	—	—	—	—	—	—	—	—
*Anachis/Costoanachis* sp.	Dovesnail	—	1	—	—	—	—	—	—	—	—	—	—	—	—	—
*Anachis/Costoanachis semiplicata*	Gulf dovesnail	5	—	—	—	1	—	—	7	3	—	4	1	1	17	79
*Anachis/Costoanachis sparsa*	Sparse dovesnail	—	—	—	—	—	—	—	—	—	—	—	—	—	6	—
*Columbella rusticoides*	Rusty dovesnail	—	—	—	—	—	—	—	—	1	—	—	—	—	—	—
*Mitrella lunata*	Lunar dovesnail	—	—	—	—	—	—	—	1	—	—	—	—	—	—	—
*Mitrella ocellata*	White-spot dovesnail	—	—	2	—	—	—	—	—	—	—	—	—	—	—	—
*Mitrella* sp.	Dovesnail	—	—	—	—	1	—	—	—	—	—	—	—	—	—	—
*Nitidella nitida*	Glossy dovesnail	—	—	—	—	—	—	—	—	—	—	—	—	—	2	2
*Suturoglypta iontha*	Lineate dovesnail	—	—	—	—	—	—	—	—	—	—	—	—	—	1	1
Columbellidae	Dovesnail	2	—	—	1	—	—	2	—	1	—	—	—	—	3	—
*Cantharus cancellaria*	Cancellate cantharus	—	—	—	—	—	—	—	—	—	—	2	—	—	2	—
*Busycon sinistrum*	Lightning whelk	78	1	305	154	131	15	649	105	261	93	340	206	316	228	205
*Busycotypus spirata*	Pearwhelk	23	1	88	40	117	2	379	167	70	22	50	30	88	117	36
*Melongena corona*	Crown conch	48	5	699	428	372	25	934	181	112	49	40	8	50	126	50
*Geomphus tinctus*	Tinted cantharus	—	—	—	—	—	—	—	—	—	—	—	—	—	2	1
*Hesperisternia multangulus*	Ribbed cantharus	—	—	—	—	—	—	—	—	—	—	—	—	—	1	—
*Nassarius/Phrontis vibex*	Bruised nassa	5	—	1	—	—	—	3	18	18	1	11	18	5	70	243
*Nassarius* sp.	Nassa	4	—	—	2	—	—	—	—	—	—	—	—	—	—	—
*Fasciolaria lilium hunteria*	Banded tulip	19	—	101	1	14	1	111	106	15	—	—	9	4	18	9
*Fasciolaria tulipa*	True tulip	1	—	3	6	4	—	15	6	7	—	10	7	3	17	3
*Fasciolaria* spp.	Tulip	1	1	37	61	7	2	—	—	10	8	—	—	—	—	—
*Pleuroploca gigantea*	Horse conch	1	—	—	1	—	—	—	2	1	1	—	—	1	3	—
*Oliva sayana*	Lettered olive	—	—	—	—	—	—	—	—	—	1	—	—	—	—	—
*Olivella floralia*	Common rice olive	—	—	—	—	—	—	—	—	—	—	—	14	—	8	22
*Olivella pusilla*	Tiny dwarf olive	—	—	—	—	—	—	—	—	—	—	—	12	—	—	—
*Olivella* spp.	Olive	2	—	1	—	3	—	—	4	3	—	5	—	—	—	—
*Granulina hadria*	Hadria marginella	—	—	—	—	—	—	—	—	—	—	—	4	—	—	—
*Marginella carnea*	Orange marginella	1	—	—	—	—	—	—	1	—	—	—	—	—	—	—
*Marginella* spp.	Marginella	15	—	1	—	1	1	3	1	5	—	—	—	—	—	—
*Prunum apicinum*	Common Atlantic marginella	—	—	—	—	—	—	—	—	—	—	—	27	2	36	101
*Prunum succinea*	Velie marginella	—	—	—	—	—	—	—	—	—	—	—	—	—	1	—
*Conus anabathrum*	Florida cone	—	—	—	—	—	—	—	—	—	—	—	—	—	4	—
*Conus* (cf.) *anabathrum*	Florida cone	—	—	—	—	—	—	—	—	—	—	—	1	—	—	—
*Conus* spp.	Cone snail	—	—	—	1	—	—	—	2	2	—	—	—	—	—	5
*Crassispira/Pyrgospira tampanensis*	Tampa turrid	2	—	—	—	—	—	—	—	—	—	—	—	—	1	—
*Crassispira* sp.		—	—	—	—	2	—	—	5	1	—	—	—	—	—	—
*Pilsbryspira leucocyma*	White-knob drilla	—	—	—	—	—	—	—	—	—	—	—	3	—	21	20
Turridae	turrid	—	—	—	—	1	—	—	—	1	—	—	—	—	—	—
*Agathotoma candidissima*	Sugar mangelia	—	—	—	—	—	—	—	—	—	—	—	7	—	6	22
*Boonea impressa*	Impressed odostome	20	—	2	33	1	—	24	30	7	—	2	5	—	2	5
*Eulimastoma canaliculatum*	Channeled odostome	—	—	—	—	—	—	—	—	—	—	—	1	—	—	—
*Odostomia laevigata*	Odostome	6	—	—	—	—	—	—	—	—	—	—	—	—	2	—
*Odostomia* sp.	Odostome	—	—	—	—	3	—	—	—	—	—	—	4	—	—	4
*Sayella fusca*	Brown sayella	—	—	—	—	—	—	—	—	—	—	—	1	—	—	—
*Turbonilla* sp.	Turbonille	—	—	—	—	1	—	—	—	—	—	—	—	—	—	—
*Turbonilla* spp.	Turbonille	—	—	—	—	—	—	—	—	—	—	—	—	—	5	2
*Pyramidellidae* spp.	Pyramid shells	—	—	—	—	—	—	—	—	—	—	—	—	—	—	4
*Bulla striata*	Striate bubble	1	—	—	—	—	—	2	1	1	—	—	3	—	1	—
*Atys* spp.	Glassy bubble	—	—	—	—	2	—	—	—	—	—	—	—	—	—	—
Atyidae	Glassy bubble	—	—	—	—	—	—	—	1	—	—	—	—	—	—	—
Aplysiidae	Seahare	2	—	—	—	—	—	—	—	—	—	—	—	—	—	—
*Acteocina canaliculata*	Channeled barrel-bubble	—	—	—	—	—	—	—	—	—	—	—	96	1	108	186
*Blauneria helerodita*	Left-hand melampus	1	—	—	—	—	—	—	—	—	—	—	1	—	—	—
*Ellobium* sp.	White melampus	—	—	—	—	—	—	—	—	—	—	—	—	—	1	—
*Melampus bidentatus*	Eastern melampus	106	—	—	3	—	—	—	2	—	—	—	33	13	55	46
*Melampus bullaoides*	Bubble melampus	—	—	—	—	—	—	—	—	—	—	—	1	—	—	—
*Melampus coffeus*	Coffee melampus	24	—	—	—	—	—	2	—	—	—	—	—	9	1	47
*Melampus* sp.	Melampus	183	1	—	—	2	—	—	—	1	—	1	—	—	—	—
*Microtralia occidentalis*	Tiny melampus	—	—	—	—	—	—	—	—	—	—	—	1	—	—	—
*Microtralia* spp.		—	—	—	—	10	—	—	—	—	—	—	—	—	—	—
*Pedipes mirabilis*	Miraculous pedipes	—	—	—	—	—	—	—	—	—	—	—	—	—	1	—
*Gastrocopta contracta*	Bottleneck snaggletooth	—	—	—	—	2	—	—	—	—	—	—	—	—	—	—
*Subulina octona*	Miniature awlsnail	—	—	—	—	—	—	—	—	3	—	—	—	—	—	—
*Huttonella bicolor*	Two-toned gulella	—	—	—	—	—	—	—	—	1	—	—	—	—	—	—
*Helicodiscus singleyanus*	Smooth coil	—	—	—	—	17	—	—	—	—	—	—	—	—	—	—
*Hawaiia miniscula*	Minute gem	—	—	—	—	5	—	—	—	—	—	—	—	—	—	—
*Euglandina rosea*	Rosy wolfsnail	—	—	—	1	—	—	—	2	—	—	1	—	—	—	—
*Polygyra cereolus*	Liptooth	85	—	—	—	—	—	—	—	—	—	—	45	1	3	—
*Polygyra* sp.	Liptooth snails	—	3	3	—	8	—	18	134	113	—	—	—	—	—	—
Polygyridae	Polygyrid land snails	—	1	—	—	—	—	—	—	—	—	10	—	—	—	—
Gastropoda	Gastropods	—	—	—	—	—	—	—	1	3	—	—	—	—	—	—
*Brachidontes exustus*	Scorched mussel	—	—	22	—	1	—	—	30	1	—	—	—	1	5	34
*Geukensia demissa*	Ribbed mussel	90	—	492	8	1160	2	175	75	82	15	79	—	—	—	—
*Geukensia granosissima*	Southern ribbed-mussel	—	—	—	—	—	—	—	—	—	—	—	2	23	30	10
*Modiolus americanus*	American horsemussel	—	—	—	—	—	—	—	—	—	—	—	—	1	1	16
Mytilidae	Mussels	—	1	3	—	—	—	—	—	—	—	—	13	—	2	—
*Anadara transversa*	Transverse ark	98	—	3	—	—	—	—	4	3	—	—	1	2	4	6
*Anadara* sp.	Ark clams	1	—	—	—	—	—	—	—	1	—	—	—	—	—	—
*Barbatia* sp.	Bearded ark clam	1	—	—	—	—	—	—	1	—	—	—	—	—	—	—
*Noetia ponderosa*	Ponderous ark	5	1	—	—	—	—	—	1	—	3	—	1	1	2	4
*Glycymeris* sp.	Bittersweet	1	—	—	—	—	—	—	—	—	—	—	—	—	—	—
*Atrina* sp.	Pens shell	—	—	—	—	—	—	—	—	—	—	—	—	1	2	1
Pinnidae	penshell	1	—	—	1	—	—	—	3	1	—	1	—	—	—	—
*Argopecten gibbus*	Atlantic calico scallop	—	—	—	—	—	—	—	—	—	—	—	—	—	1	—
*Argopecten irradians*	Bay scallop	2	—	1	1	1	2	2	37	3	—	3	—	1	3	3
*Plicatula gibbosa*	Atlantic kittenpaw	4	—	—	—	—	—	—	—	—	—	—	—	—	—	7
*Anomia simplex*	Common jingle	2	—	2	—	—	—	—	6	2	—	—	—	3	29	60
*Crassostrea virginica*	Eastern oyster	240	18	265	309	76	4	194	196	176	71	675	462	312	236	118
*Ostreola equestris*	Crested oyster	97	—	48	5	5	—	22	190	25	3	33	25	34	42	170
Ostreidae	oyster	—	—	48	—	—	—	—	—	—	—	51	—	—	—	—
*Codakia orbicularis*	Tiger lucine	—	—	—	—	—	—	—	—	1	—	—	—	—	—	—
*Codakia orbiculata*	Dwarf tiger lucine	—	—	—	—	—	—	—	—	2	—	—	—	—	—	—
*Lucina nassula*	Woven lucine	—	—	—	—	—	—	3	—	—	—	—		2	2	2
*Radiolucina amianta*	Miniature lucine	—	—	—	—	—	—	—	—	—	—	—	1	—	—	—
*Stewartia floridana*	Florida lucine	—	—	—	—	—	—	—	—	—	—	—	1	—	—	—
Lucinidae	Lucine	—	—	—	—	—	—	—	—	—	—	—	—	—	—	3
*Carditamera floridana*	Broad-ribbed carditid	7	5	4	1	—	—	2	9	6	—	4	1	4	—	18
*Crassinella lunulata*	Lunate crassinella	1	—	—	—	—	—	—	1	—	—	—	—	—	—	—
*Crassinella martinicensis*	Martinique crassinella	—	—	1	—	—	—	—	—	—	—	—	—	—	—	—
*Crassinella (*cf.) *lunulata*	Lunate crassinella	—	—	—	—	—	—	—	—	—	—	—	2	—	—	4
*Dinocardium robustum*	Giant Atlantic cockle	—	1	1	1	—	—	—	—	—	1	—	—	1	1	1
*Trachycardium egmontianum*	Florida prickly cockle	2	—	—	—	—	—	—	—	—	—	—	—	—	—	1
*Trachycardium muricatum*	Yellow prickly cockle	—	—	—	—	—	—	—	—	—	—	—	—	—	—	1
*Trachycardium* sp.	Prickly cockle	—	—	—	—	1	—	—	—	—	—	—	—	—	—	—
Cardiidae	Cockles	—	—	—	—	—	—	1	—	—	—	—	—	—	—	—
Pectinidae/Cardiidae	Scallops/cockles	—	—	—	—	—	1	—	—	—	1	—	—	—	—	—
*Spisula solidissima*	Atlantic surf clam	19	87	16	1	—	—	—	6	2	1	12	—	—	—	—
*Spisula raveneli*	Southern surf clam	—	—	—	—	—	—	—	—	—	—	—	2	—	5	2
*Spisula* sp.	Surf clam	—	—	—	—	—	—	—	—	—	—	—	—	1	—	—
*Macoma* spp.	Macoma	—	—	—	—	—	—	3	—	—	—	—	—	—	—	—
*Tampaella tampaensis*	Tampa tellin	—	—	—	—	—	—	—	—	—	—	—	8	12	—	155
*Tellina lineata*	Rose-petal tellin	—	—	—	—	—	—	—	—	—	1	—	—	—	—	—
*Tellina* sp.	Tellin	—	—	2	—	3	—	—	2	—	—	—	—	—	2	—
Tellinidae	Tellin	—	—	—	—	—	—	—	—	1	—	—	—	—	—	—
*Donax variabilis*	Variable coquina	9	1	1	—	—	—	—	3	1	—	—	—	1	2	2
*Tagelus* sp.	Tagelus	—	—	—	—	—	—	—	—	—	—	—	—	—	—	6
*Cumingia coarctata*	Contracted semele	—	—	—	—	—	—	—	—	—	—	—	—	—	1	—
*Semele proficua*	Atlantic semele	—	—	—	—	—	—	—	—	—	—	—	—	—	1	—
*Semele* sp.	Semele	3	1	—	—	—	—	—	—	—	—	—	—	—	—	—
*Polymesoda carolina*	Carolina marshclam	—	—	—	2	—	—	—	—	—	—	—	—	—	—	—
*Polymesoda floridana/maritima*	Southern marshclam	1137	—	28	12	21	—	24	18	21	—	—	57	29	232	945
*Arcinella cornuta*	Florida spiny jewelbox	—	—	—	—	—	—	—	—	—	—	—	—	—	—	1
*Diplodonta* sp.	Diplodonta	—	—	—	—	—	—	—	—	—	—	—	—	—	1	—
*Anomalocardia auberiana*	Pointed venus	21	—	12	2	5	—	13	2	—	—	3	63	47	184	1740
*Anomalocardia* sp.		—	—	—	2	—	—	—	—	—	—	—	—	1	2	12
*Chione cancellata*	Cross-barred venus	9	—	2	62	1	—	—	1	1	—	4	—	—	—	—
*Chione grus*	Gray pygmy-venus	2	—	—	—	1	—	—	1	—	—	—	—	—	—	—
*Chione* sp.	Venus clam	1	—	—	—	—	—	—	—	—	—	—	—	—	—	—
*Macrocallista nimbosa*	Sunray venus	—	—	—	11	—	—	—	1	—	1	13	—	—	—	—
cf. *Macrocallista nimbosa*	Sunray venus	—	—	—	—	1	—	—	—	—	—	—	—	—	1	1
*Mercenaria campechiensis*	Southern quahog	3	5	1	8	2	3	2	2	2	4	1	—	—	1	5
Veneridae	Venus clam	1	—	—	—	—	—	1	—	—	—	—	—	—	—	—
*Transennella conradina*	Conrad’s transennella	—	—	17	—	24	—	—	—	—	—	—	53	33	108	237
*Transennella* spp.	Transennella	—	—	—	—	—	—	25	—	—	—	—	—	—	—	—
*Corbula* sp.	Corbula	—	—	—	—	—	—	—	—	1	—	—	—	—	—	—
*Parastarte triquetra*	Brown gemclam	10	—	24	1	42	—	13	18	16	—	—	345	4	254	720
Bivalvia	bivalves	—	—	6	—	8	—	—	—	—	—	—	—	—	—	—
Brachiopoda	Lamp shells	1	—	—	—	—	—	—	—	—	—	—	—	—	—	—
Anthozoa	Hard corals	1	—	—	—	—	—	—	1	1	—	1	—	—	—	—
Xanthidae	Mud crabs	1	—	2	—	—	—	—	—	—	—	—	—	—	—	—
*Callinectes* spp.	Swimming crab	—	—	5	2	1	—	—	—	—	—	—	—	—	—	—
*Callinectes sapidus*	Blue crab	1	—	—	—	—	—	—	—	—	—	—	—	—	—	—
*Menippe mercenaria*	Stone crab	—	—	—	—	—	—	—	—	—	—	1	—	—	—	—
Majidae cf. *libinia*	Spider crab	—	—	3	—	1	—	—	—	—	—	—	—	—	—	—
Decapoda	crabs, shrimp, lobsters	—	—	—	—	—	—	3	—	2	—	—	—	—	—	—
Brachyura	True crabs	—	—	2	1	1	—	—	2	—	—	—	—	—	—	—
*Balanus eburneus*	Ivory barnacles	—	—	—	—	—	—	—	—	—	—	17	—	—	—	—
*Balanus* spp.	Acorn barnacles	—	—	509	—	52	—	—	—	—	—	—	55	1	188	88
*Chthamalus fragilis*	Fragile barnacle	—	—	1	—	2	—	—	—	—	—	—	—	—	—	—
Cirripedia	barnacle	163	2	—	115	—	—	152	22	35	—	—	—	—	—	—
*Echinoidea* cf. *clypeaster*	Sea biscuit	1	—	—	—	—	—	—	—	—	—	—	—	—	—	—
Echinoidea	Sea urchin	4	4	1	—	1	1	1	2	1	—	3	—	—	—	—
*Carcharhinus* cf. *acrinotus*	Blacknose shark	—	—	—	—	—	1	—	—	—	—	—	—	—	—	—
*Carcharhinus leucus*	Bull shark	—	—	—	1	—	—	—	—	—	—	—	—	—	—	—
*Carcharhinus limbatus*	Blacktip shark	—	1	1	—	—	—	—	—	2	—	—	—	—	—	—
*Carcharhinus limbatus/brevipinna*	Blacktip/spinner shark	—	—	—	2	—	—	—	—	—	—	—	—	—	—	—
*Carcharhinus* sp.	Requium shark	—	—	—	—	—	—	—	—	—	—	—	—	—	1	—
*Carcharhinus* spp.	Requium shark	1	—	—	—	—	—	—	—	—	—	1	—	—	—	—
*Galeocerdo cuvier*	Tiger shark	—	—	—	1	—	—	—	—	—	1	—	—	—	—	—
*Negaprion brevirostris*	Lemon shark	—	—	—	—	—	—	—	—	1	1	1	—	—	—	—
*Rhizoprionodon terraenovae*	Atlantic sharpnose shark	1	—	1	—	—	—	—	—	—	—	1	—	—	—	—
*Sphyrna lewini*	Scalloped hammerhead	—	—	—	—	—	1	—	—	—	—	—	—	—	—	—
*Sphyrna mokarram*	Great hammerhead	—	—	—	—	—	—	—	1	—	—	—	—	—	—	—
*Sphyrna tiburo*	Bonnethead shark	—	—	1	—	—	—	—	1	1	1	—	—	—	—	—
Sphyrnidae	Hammerhead sharks	—	—	—	—	—	—	—	—	—	—	—	—	—	—	1
*Pristis* sp.	Sawfish	—	—	—	—	—	—	—	—	—	—	1	—	—	—	—
*Rhinobatus* sp.	Guitarfish	—	—	—	1	—	—	—	—	—	—	—	—	—	—	—
*Dasyatis americana*	Southern stingray	—	—	—	—	—	1	—	—	—	—	—	—	—	—	—
*Dasyatis* sp.	Stingray	—	—	—	1	1	—	—	—	1	1	—	—	—	—	—
Dasyatidae	Whiptail stingrays	—	—	—	—	—	—	—	—	—	—	1	—	—	1	1
Myliobatidae	Eaglerays	—	—	—	—	—	—	—	—	—	—	—	—	—	1	—
*Rhinoptera bonasus*	Cownose ray	—	—	1	—	—	—	—	—	—	1	—	—	—	—	—
Rajiformes	rays, skates	2	—	—	—	—	—	1	1	—	—	—	1	—	1	1
Chondrichthyes	Cartilagenous fishes	—	—	—	—	—	—	—	—	—	—	—	—	1	—	—
*Amia calva*	Bowfin	—	—	—	—	—	—	—	—	—	—	—	—	—	—	1
*Lepisosteus* sp.	Gar	1	—	1	—	—	—	—	—	—	1	—	—	—	—	—
*Elops saurus*	Ladyfish	—	1	—	—	—	—	1	1	1	—	1	—	—	—	—
Elopidae	tarpons, ladyfish	—	—	—	—	—	1	—	—	—	—	—	—	—	—	—
*Brevoortia smithi*	Menhaden	—	—	—	—	—	—	—	—	—	—	1	—	—	—	—
*Harengula humeralis*	Redear sardine	—	—	—	—	—	1	—	—	—	—	—	—	—	—	—
*Harengula* spp.	Sardine	—	—	—	—	—	—	—	—	5	2	—	—	—	—	—
*Opisthonema oglinum*	Threadfin	12	—	5	—	—	—	—	7	25	12	38	—	—	—	—
Clupeidae	herring	1	3	2	2	1	1	1	—	1	—	—	—	—	1	—
*Ictalurus* sp.	Freshwater catfish	—	—	—	—	—	—	—	—	—	—	1	—	—	—	—
*Arius felis*	Hardhead catfish	5	1	3	15	4	14	7	6	25	27	13	—	—	4	—
*Bagre marinus*	Gafftopsail	1	—	—	1	—	1	—	—	2	3	1	—	—	—	—
Ariidae	Sea catfishes	—	1	1	—	—	5	—	—	—	—	—	6	13	—	9
*Synodus* sp.	Lizardfish	—	—	—	1	—	—	—	—	—	—	—	—	—	—	—
*Opsanus* spp.	Toadfish	1	1	2	4	1	—	1	3	2	1	3	1	—	1	2
*Chriodorus atherinoides*	Hardhead halfbeak	1	—	1	—	—	—	—	—	—	—	—	—	—	—	—
Exocoetidae	Flyingfish and halfbeaks	1	—	—	—	—	—	1	—	—	—	—	—	—	—	—
*Strongylura* sp.	Needlefish	1	—	—	2	—	—	—	—	—	—	—	—	—	—	—
*Tylosurus* sp.	Houndfish	1	—	—	—	—	—	—	—	—	—	—	—	—	—	—
cf. *Tylosurus* sp.	Houndfish	—	—	—	—	1	—	—	—	—	—	—	—	—	—	—
Belonidae	needlefish	—	1	1	—	1	1	3	2	2	1	3	—	—	—	—
*Fundulus* spp.	Killifish	3	—	3	16	3	1	5	2	5	2	8	—	—	—	—
Cyprinodontidae	Killifishes	—	—	—	—	—	1	—	—	—	—	—	—	—	—	—
Atherinidae	Silversides	—	—	—	—	—	—	—	—	1	—	—	—	—	—	—
*Centropomus undecimalis*	Common snook	—	—	—	—	—	—	—	—	—	1	—	—	—	—	—
*Centropomus* sp.	Snook	—	—	—	—	—	—	—	—	1	—	—	—	—	—	—
Centropomidae	snook	—	—	—	—	—	—	—	—	—	—	1	—	—	—	—
*Prionotus* sp.	Searobin	—	—	—	—	—	—	—	—	1	—	—	—	—	—	—
*Epinephelus* sp.	Grouper	1	1	—	1	—	—	—	1	—	1	1	—	—	1	—
Serranidae	Groupers and seabasses	—	—	1	—	—	—	—	—	—	—	—	—	—	—	2
*Caranx crysos*	Blue runner	—	—	—	—	—	—	—	—	1	—	—	—	—	—	—
*Caranx crevalle/hippos*	Crevalle jackfish	—	—	—	—	—	—	—	—	—	1	—	—	—	—	—
*Caranx latus*	Horse-eye jack	—	—	—	—	—	—	—	—	—	—	—	—	—	2	—
*Caranx* sp.	Jacks	1	—	—	1	1	1	—	2	2	1	1	—	—	—	—
*Chloroscombrus chrysurus*	Atlantic bumper	—	—	—	—	—	—	—	—	—	2	2	—	—	—	—
*Selene vomer*	Lookdown	—	—	—	—	—	—	—	2	3	—	—	—	—	—	—
Carangidae	Jacks	—	—	—	—	—	—	—	—	—	—	2	—	—	—	—
*Lutjanus* sp.	Snapper	1	—	—	—	1	—	—	1	2	—	—	—	—	—	—
*Lutjanus* spp.	Snappers	—	—	—	—	—	—	—	—	—	—	—	—	—	2	—
Lutjanidae	Snappers	—	—	—	—	—	—	—	—	—	—	1	—	—	—	1
*Eucinostomus gula*	Silver jenny	—	—	1	—	—	—	—	—	—	—	—	—	—	—	—
*Eucinostomus* spp.	Mojarra, jenny	4	—	—	2	2	7	—	10	3	4	2	—	—	—	—
Gerreidae	Mojarra, jenny	—	—	—	—	—	—	—	—	—	—	—	—	—	1	—
*Anisotremus* sp.	Porkfish	—	—	—	—	—	—	—	—	1	—	—	—	—	—	—
*Haemulon* sp.	Grunt	—	—	—	—	—	—	—	—	—	—	1	1	—	3	—
*Orthopristis chrysoptera*	Pigfish	11	—	3	17	6	24	1	27	48	13	38	—	—	—	1
cf. *Orthopristis*	Pigfish	—	—	1	—	—	—	—	—	—	—	—	—	—	—	—
Haemulidae	grunts	—	—	—	—	—	—	—	—	—	—	1	—	—	—	—
*Archosargus probatocephalus*	Sheepshead	2	1	—	3	—	2	2	1	3	1	2	—	—	—	1
*Diplodus holbrookii*	Spottail pinfish	—	—	—	—	—	—	—	—	—	—	—	—	—	1	—
*Lagodon rhomboides*	Pinfish	69	4	22	122	20	93	8	55	176	79	162	—	—	1	3
*Pagrus pagrus*	Red porgy	—	—	—	—	—	—	—	—	—	—	—	—	—	1	—
Sparidae	Porgies	—	—	1	—	—	—	—	—	—	7	—	2	—	—	—
*Bairdiella chrysoura*	Silver perch	3	—	3	2	—	—	—	5	19	6	14	1	—	2	1
*Cynoscion arenarius*	Sand seatrout	—	—	—	—	—	—	—	—	—	—	1	—	—	—	—
*Cynoscion nebulosus*	Spotted seatrout	—	—	1	—	—	—	2	—	—	6	3	1	1	—	7
*Cynoscion* sp.	Seatrout	2	2	1	6	1	2	1	3	7	—	—	1	5	2	—
*Leiostomus xanthurus*	Spot	23	—	—	1	1	—	—	4	6	1	1	—	—	—	—
*Menticirrhus saxatilis*	Northern Kingfish	—	—	—	—	—	—	—	—	—	—	—	—	—	—	1
*Menticirrhus* sp.	Kingfish	1	—	—	—	—	—	—	1	—	—	—	—	—	—	—
*Pogonias cromis*	Black drum	—	—	—	—	—	—	—	—	—	—	1	—	—	1	—
*Sciaenops ocellatus*	Red drum	1	—	—	—	—	—	1	—	6	3	3	1	2	1	2
Sciaenidae	Drum fishes	—	—	—	—	1	—	—	—	—	—	—	—	—	—	—
Mullidae	Goatfishes	—	—	—	—	—	—	—	1	—	—	—	—	—	—	—
*Mugil* spp.	Mullet	2	1	2	4	1	1	3	2	2	1	2	1	1	3	3
*Sphyraena* sp.	Barracuda	—	—	—	—	—	1	—	—	—	—	—	—	—	—	—
*Gobiomorus dormitor*	Bigmouth sleeper	—	—	—	—	—	1	—	—	—	—	1	—	—	—	—
*Paralichthys lethostigma*	Southern flounder	—	—	—	—	—	—	—	—	—	—	1	—	—	—	—
*Paralichthys* sp.	Flounder	—	—	—	—	—	—	—	—	—	—	—	—	—	1	—
*Paralichthy*s spp.	Flounder	3	—	—	2	—	1	—	1	3	1	—	—	—	—	—
Bothidae	Left-handed flatfish	—	—	2	—	—	—	1	—	—	—	2	—	1	—	—
*Lactophrys* sp.	Trunk fish	—	—	—	—	—	—	—	—	—	—	1	—	—	1	—
*Chilomycterus schoepfi*	Striped burrfish	—	—	3	—	2	1	—	—	—	1	3	2	—	—	—
*Chilomycterus* spp.	Burrfish	2	—	—	3	—	—	4	—	3	—	—	—	—	—	—
*Diodon* sp.	Porcupinefish	—	—	—	—	—	—	—	1	—	—	—	—	—	—	—
*Diodon* spp.	Porcupinefish	—	—	—	—	—	—	—	—	—	—	—	1	1	2	—
Diodontidae	Burr and porcupine fish	—	—	—	—	—	—	—	—	—	—	—	—	—	—	1
*Lagocephalus* sp.	Puffer	—	—	—	1	—	—	—	—	—	—	—	—	—	—	—
*Spheroides* sp.	Puffer	—	—	—	—	—	1	—	2	3	1	3	—	—	—	—
cf. *Spheroides*	Puffer	—	—	1	—	1	—	1	—	—	—	—	—	—	—	—
Tetraodontidae	puffer/burrfish	—	2	—	—	—	—	—	—	—	—	—	—	—	—	—
Osteichthyes Species A	Bony fish Species A	—	—	1	2	3	1	—	1	4	1	1	—	—	—	—
*Amphiuma* sp.	Amphiuma	—	—	—	—	—	—	—	—	1	—	—	—	—	—	—
Anura	Frogs	1	—	—	—	—	—	—	—	1	—	—	—	—	—	—
Colubridae	Nonvenomous snakes	3	—	—	—	—	—	—	—	1	1	—	—	—	1	—
Viperidae	Venomous snakes	1	—	—	—	—	—	—	—	—	—	—	—	—	—	—
Serpentes	Snakes	—	—	—	—	—	—	—	1	—	—	—	—	—	—	—
*Anolis* sp.	Anole lizard	—	—	—	—	—	—	—	—	1	—	—	—	—	—	—
Lacertilia	Lizards	—	—	—	2	—	—	—	1	—	—	1	—	—	—	—
*Chelydra serpentina*	Snapping turtle	—	—	—	—	—	—	—	1	—	—	—	—	—	—	—
Chelydridae	Snapping turtles	—	—	—	—	—	—	—	—	1	—	—	—	—	—	1
*Malaclemys terrapin*	Diamondback terrapin	—	—	—	—	1	—	—	—	—	—	—	—	1	—	—
*Trachemys* sp. */Pseudemys* sp.	Sliders, cooters	—	—	—	—	—	—	—	—	—	—	—	—	—	—	1
*Kinosternon* sp.	Mud turtles	—	—	—	—	—	—	1	1	—	—	—	—	—	—	—
*Sternotherus* sp.	Musk turtles	1	—	—	—	—	—	—	—	—	—	—	—	—	—	—
Cheloniidae	Sea turtles	2	—	—	—	—	—	—	—	—	—	—	—	—	—	—
*Gopherus polyphemus*	Gopher tortoise	1	—	—	—	—	—	—	—	—	—	—	—	—	1	1
*Terrapene carolina*	Eastern box turtle	—	—	—	—	—	—	—	—	—	—	1	—	—	—	—
Testudines	Turtles	—	2	1	2	—	1	—	—	1	—	—	—	—	—	—
*Larus atricilla*	Laughing gull	—	—	1	—	—	—	—	—	—	—	—	—	—	—	—
Rallidae	Rails	—	—	—	—	—	1	—	—	—	—	—	—	—	—	—
*Gavia immer*	Common loon	—	—	—	1	—	—	—	—	—	—	—	—	—	—	—
Aythyinae	Sea ducks	—	—	—	—	—	—	—	—	—	1	—	—	—	—	—
Anatidae	Ducks, geese, swans	1	—	—	10	1	—	—	1	1	—	—	—	—	—	—
Aves	Birds	—	—	—	—	—	—	1	—	—	—	1	1	—	1	1
*Oryzomys palustris*	Rice rat	—	—	—	—	—	—	—	—	1	—	—	—	—	—	—
*Sigmodon hispidus*	Hispid cotton rat	1	—	—	1	—	—	—	—	1	1	1	—	—	—	—
Cricetidae	New World Rodents	—	—	—	—	—	1	—	—	—	—	—	—	—	—	—
Rodentia	Rodent	—	—	1	—	—	—	1	—	1	—	—	—	—	—	1
*Procyon lotor*	Raccoon	—	—	1	—	—	—	—	—	—	—	—	—	—	1	1
*Odocoileus virginianus*	White-tailed deer	1	—	1	—	—	1	—	—	1	1	1	—	—	—	1
Cetacean	Aquatic mammals	—	—	—	—	—	—	—	—	—	—	—	—	—	—	1
Mammalia	Mammals	—	1	—	1	—	—	—	—	—	—	—	—	1	—	—
Vertebrata	Vertebrates	—	—	—	—	1	—	—	—	—	—	—	—	—	—	—
**Totals**		**3480**	**164**	**2957**	**1561**	**2241**	**230**	**3293**	**1762**	**1518**	**479**	**1797**	**1949**	**1156**	**2631**	**9949**

*Where original analyses had multiple species A, B, C in other analysis they were combined to family

Each archaeological context reported (see Table 2) is used as a separate locale to investigate spatial and temporal patterns based on species presence and abundance. Further, to investigate a model of environmental change based on the data included here, all identified invertebrate taxa were given environment (habitat) designations by roughly segmenting the Charlotte Harbor/Pine Island Sound region into different salinity regimes as they are present today following Holland-Lulewicz and Thompson [36]. These habitat classifications are tidal stream, estuarine mangrove, oyster bed, seagrass meadow, and littoral zone. First, we focus on the analysis of a single stratigraphic unit, Operation P reported in Holland-Lulewicz and Thompson [36]. Second, we use the site-wide data to examine larger potential spatial and temporal trends.

#### Stratigraphic assemblages

For the analysis of a single, high-resolution stratigraphic column, we use materials analyzed from Operation P, a trench excavated in 2015 and 2017 [36, 49] (see Table 2). Today this location sits approximately 20 meters from the shoreline, along the southwestern portion of the Randell Complex (Fig 2). These excavations revealed a water-logged, anaerobic shell midden with well-preserved stratification indicating primary deposits of a midden that likely accumulated at the water’s edge. High-resolution AMS dating and Bayesian modeling place the deposits between AD 1000 and 1500 and lend generational-scale resolution to each arbitrary 10cm level. Zooarchaeological analyses were conducted on four of these levels: 99, 101, 103, and 105 from bulk-collected samples, each a 50cmx50cm square, 10cm thick. As stated above, analysis occurred on all invertebrate remains screened to 1/16^th^ inch (1.6mm) while and on all vertebrate remains screened to 1/8^th^ inch (3mm). MNI was calculated for each arbitrary level as a single unit.

#### Site-wide assemblages

As stated above, the Pineland Site Complex is a large site with many features and the ongoing research programs have produced ample amounts of zooarchaeological data with which to illustrate the effectiveness of network-based approaches. The zooarchaeological data used for our site-wide analysis was originally analyzed and published by deFrance and Walker [2] who target assemblages from across Pineland and analyzed all faunal remains recovered to 1/16^th^ inch (1.6mm) screen. The eleven samples analyzed by these authors, and used here, come primarily from mound contexts and each represent individual stratigraphic layers. These include two layers from Old Mound, one layer from Surf Clam Ridge, one layer from Low Mound, three layers from Brown’s Complex, two layers from Randell Complex Mound 1, and two layers from Brown’s Complex Mound 2 (see Fig 2, Table 2). These layers are representative of distinct time periods (see Table 1).

Old Mound, Surf Clam Ridge, and Low Mound contain deposits that represent some of the earliest occupation at the Pineland site during the Caloosahatchee I Late period (AD 1–500) and Caloosahatchee IIA period (AD 500–800) (Fig 2). Old Mound is a 3-m high shell midden-mound located on the southern and eastern portion of the site and is largely impacted by modern anthropogenic disturbances. The Old Mound contexts included in the analysis here come from Operation A, a 4mx4m block excavation, split into 16 1mx1m units, excavated in 10cm levels, and was designed to capture the lowest deposits including those that were waterlogged. Faunal samples were analyzed from Operation A, Unit 8, Level 101 Stratum 9/10 (Caloosahatchee I) and Operation A, Unit 16, Level 92, Stratum 3 (Caloosahatchee IIA).

Surf Clam Ridge is a long linear shell midden of approximate 125m in length and 1.5 m in height centrally located within the Pineland site, paralleling the nearby shoreline (Fig 2). Faunal samples were analyzed from Trench 11B, a backhoe trench 24.5 m in length. A 50x50x10cm bulk sample was selected to target Stratum 4 for zooarchaeological analysis of deposits dating to Caloosahatchee I. Also representative of the Caloosahatchee I Late period, Low Mound is a low sand and shell mound with a peak elevation at 2.9m. Operation A here was a 1x2m excavation unit with faunal samples analyzed from Unit 1, Level 77 from Stratum 2.

Brown’s Complex is located on the western portion of the site close to the shore of Pine Island Sound just north of the Randall Complex (Fig 2). The two complexes are bisected by the Pine Island Canal. There are 5 main features associated with Brown’s Complex but important for this discussion are Mounds 1 and 2 at heights of 9m and 5.5 m respectively. Brown’s Complex is represented by faunal samples that date to the Caloosahatchee IIA, IIB (AD 800–1200), and III (AD 1200–1350) periods. The faunal samples representative of Caloosahatchee IIA come from Operation C Units 5 and 6 both located in the swale between Mounds 1 and 2. Operation C Unit 5 was a 2x2m block in the center of the original 4x4m block and Operation C Unit 6 was excavated the following year as outward extension of Unit 5. Faunal samples from Operation C include a Level 79 50x50x10cm sample combining portions of strata 4, 6, and 16. From Operation C-6 two faunal samples were analyzed with the first from Level 88 Stratum 8 and the second from Level 91 Stratum 13. The two samples that represent the later occupation at Pineland were excavated from the summit of Brown’s Complex Mound 2 dating to Caloosahatchee IIB and III from Operation I-2, a 2x2m unit, from Level 66 Stratum 14 and Level 73 Stratum 19, respectively.

The Randell Complex is also located at the western portion of the site close to the shore of Pine Island Sound south of Brown’s Complex (Fig 2). Mound 1 is a 7m tall, large, flat-topped, rectangular, shell mound. Mound 1 suffered a truncation in the 1950s that left a portion of the mound slumped with an exposed portion that was cleaned up in order to examine the mound profile. During this profile clean-up and exposition, two faunal samples were collected from Level 55 Stratum 4 and Level 63 Stratum 7, both dating to Caloosahatchee IIB.

### Network analysis and visualization

Here we attempt to visualize zooarchaeological assemblages, and by proxy the human-centered ecosystems they reflect, by way of network graphs. Network graphs are simple, made up of nodes and ties. Traditionally, nodes represent people, institutions, communities, or other kinds of entities, whereas ties represent some kind of interaction, association, flow, or co-occurrence among entities. A tie can reflect simply the presence/absence of a connection or co-occurrence, or it can reflect a specific value (for example, the number of times two nodes interact). Finally, similar to a correspondence analysis, the distance between two nodes directly represents similarities or differences in their ties. For more information on the foundations of network analysis, a number of accessible introductions are available [50–52]. Further, a number of reviews and examples of archaeological applications of network analyses can also be consulted (e.g., 12–16), including the recently published *Network Science in Archaeology* [13]. While a review of network methods and applications, broadly and within archaeology, is beyond the scope of this paper, we do provide a walk-through of reading the networks presented here based on zooarchaeological data. Because we move from material proxies, in this case zooarchaeological data, to more abstract network visualizations, the graphs can be conceptually difficult to interpret.

**Fig 3:**
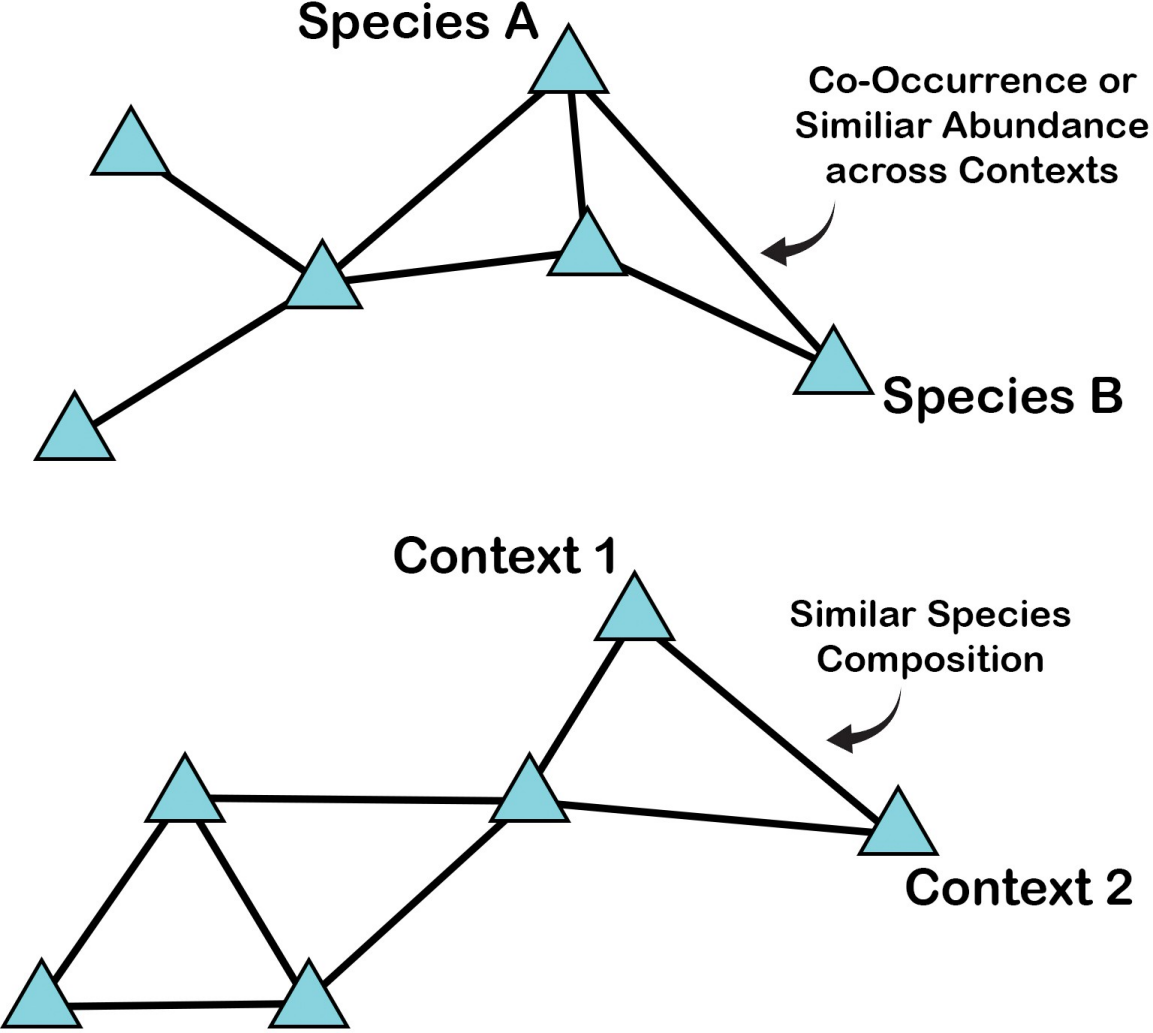
Examples of the kinds of nodes and ties used in this study to construct network graphs.

**Fig 4:**
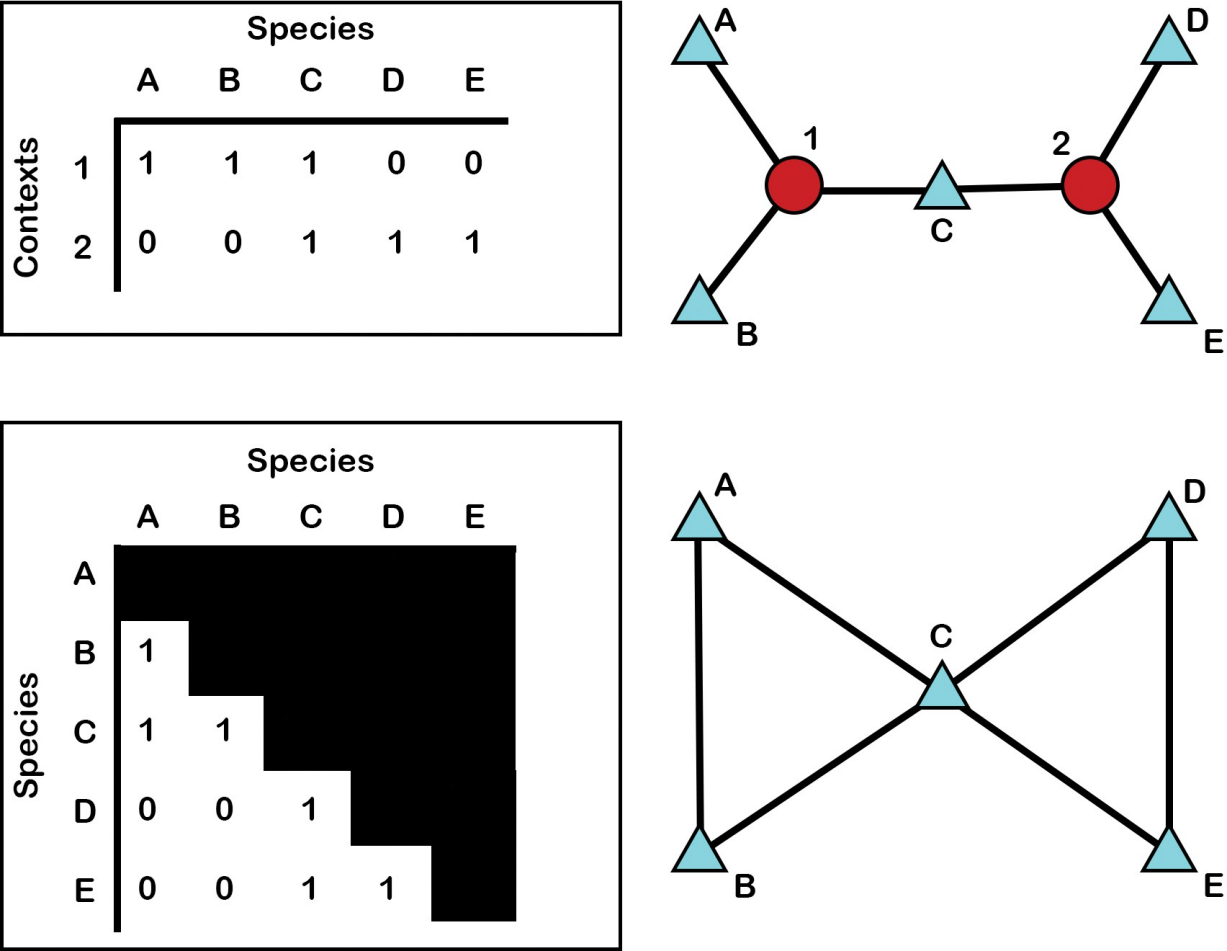
Examples of one mode (top) and two mode (bottom) networks alongside examples of matrices that underlie network graphs.

For the graphs we build here, nodes might represent specific contexts, specific species, or specific habitats (Fig 3). In some cases, ties represent the presence of a specific species in a specific context or habitat. In other cases, ties represent a certain cutoff point of percent MNI for a specific context (for example, all cases where percent MNI is over 5%). Networks are built from simple data tables, or matrices (Fig 4), such as a species list Indicating MNI or presence/absence for each species (columns) within particular contexts (rows). Another example may be a similarity matrix, where rows and columns both represent species, and the values within the matrix represent how similar those species are in regard to the contexts in which they are found.

We use two different kinds of networks: two-mode networks and one-mode networks. Two-mode networks contain two different kinds of nodes with ties between different classes of nodes (Fig 4, top). An example of this is a network depicting both contexts and species as nodes, with ties drawn between contexts and species. A one-mode network is a transformation of a two mode-network, where only a single kind of node is depicted such as a network where nodes represent only species (Fig 4, bottom). Here, ties are drawn between species representing the strength of co-occurrence of species in particular contexts. That is, we might select to draw ties between species present in 50% or more of the same contexts.

In some cases, we integrate visualizations of assemblage, species, or context characteristics into the network graphs. For instance, a node representing a single species may be colored to reflect the habitat within which it lives. A specific context (e.g., a layer within a mound) may be colored to reflect the time-period to which it can be assigned or it may be sized (e.g., bigger or smaller) to reflect its species diversity or richness.

While we do not dive deeply into any kinds of advanced analyses, metrics, or statistics, we do leverage a simple community detection algorithm to identify and visualize groups or packages of species that co-occur with one another in similar abundance across time and space. The algorithm we use is called the Girvan-Newman algorithm [53]. This algorithm is an iterative process that is used to identify cohesive subgroups within a network graph. This is also sometimes referred to as community detection. Simply put, the algorithm iteratively deletes ties between dense clusters within the network, seeking groupings that maximizes strong, within group ties and minimizes weaker, between group ties eventually revealing the most salient properties of the overall community structure of the network.

All our analyses are conducted in UCINET and its network visualization tool, NetDraw [54]. This is an easy-to-use software with an easily navigable user interface that requires no coding. The majority of relevant network metrics and processes in published literature have been written into the program. Additionally, network graph visualizations can be easily manipulated via direct point-and-click. Further accessibility to the software is provided by its companion book, which serves as both a guide to the software as well as a thorough introduction to network analyses and research design [50]. All data are imported into UCINET as Microsoft Excel files. From a species list, it is little work to bring the data into UCINET and begin visualizing network graphs. All Excel spreadsheets and their associated UCINET files are openly available at Zenodo [55]. Excel files can be imported into UCINET and converted to UCINET files, or the UCINET files can be directly downloaded and opened immediately in NetDraw. We choose to use UCINET as our example because it requires no previous knowledge of coding or any skill in using programs like R. That said, all of our data are openly accessible and all of the analyses presented here can be completed in open software, like R, as one wishes. In fact, Borgatti et al [56] have authored a book that mirrors their UCINET companion book for network analysis in R while Brughmans and Peeples’s [13] book on archaeological network science includes an extensive online component with R tutorials.

## Results and discussion

### Stratigraphic analysis

**Fig 5:**
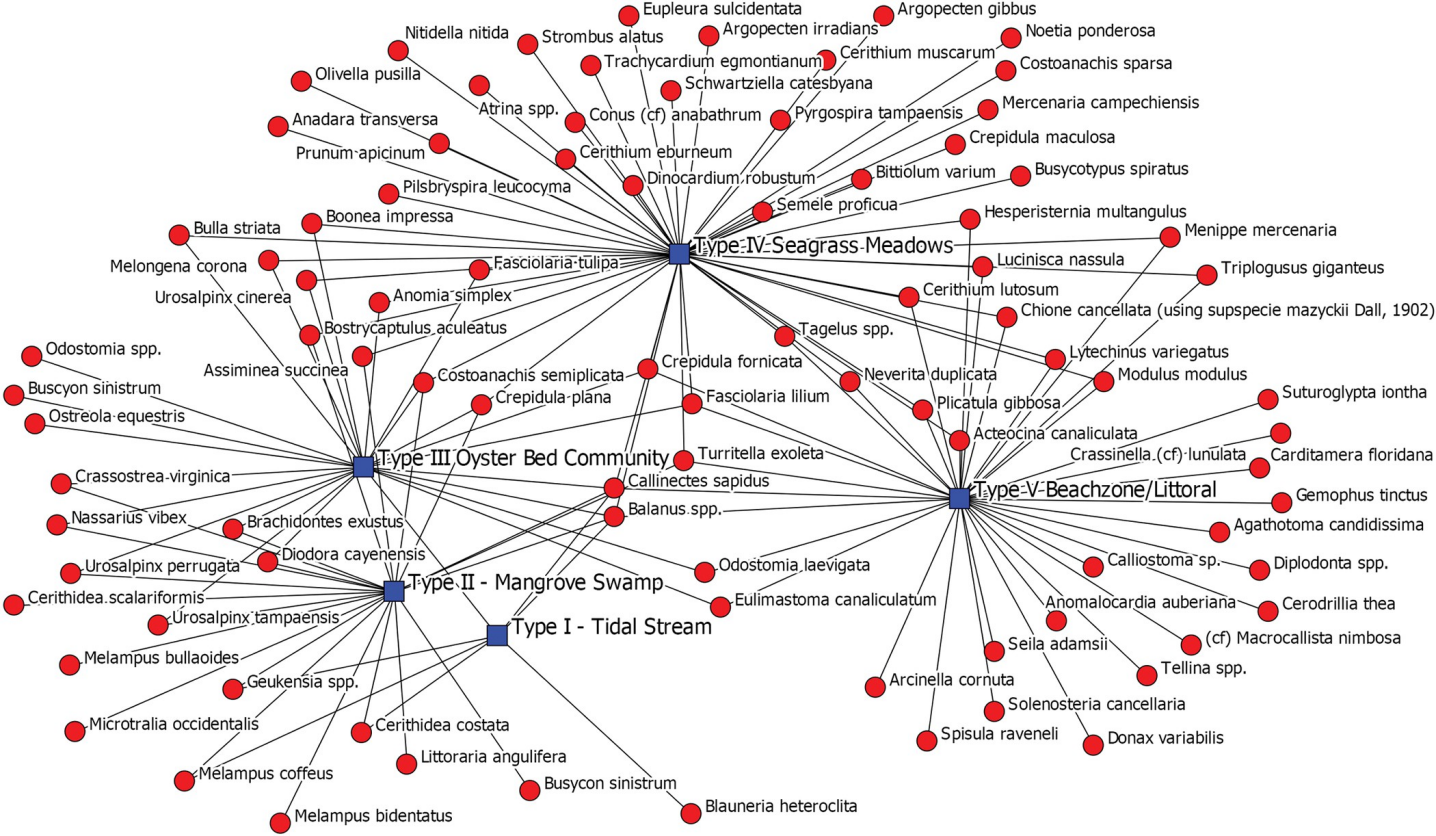
Two mode network depicting all invertebrate species found in Operation P stratigraphic layers (red circles) and the habitats that those species inhabit (blue squares). Ties represent the presence of a species in a stratigraphic layer.

The first visualization we produced for Operation P (Fig 5) is a simple graph with two kinds of nodes: the blue squares represent the five habitat categories while red circles represent invertebrate species. This is essentially an ego-network of a human-centered ecosystem highlighting human-habitat-invertebrate relationships while reflecting its composition, content, and connections as manifest in the zooarchaeological data. While not a reflection of the wider, total ecosystem, it reflects the direct ecosystem within which humans were engaged and embedded. It reflects habitats and species directly exploited, whether by purposeful capture or by-catch, or those species that colonize shell midden contexts.

**Fig 6:**
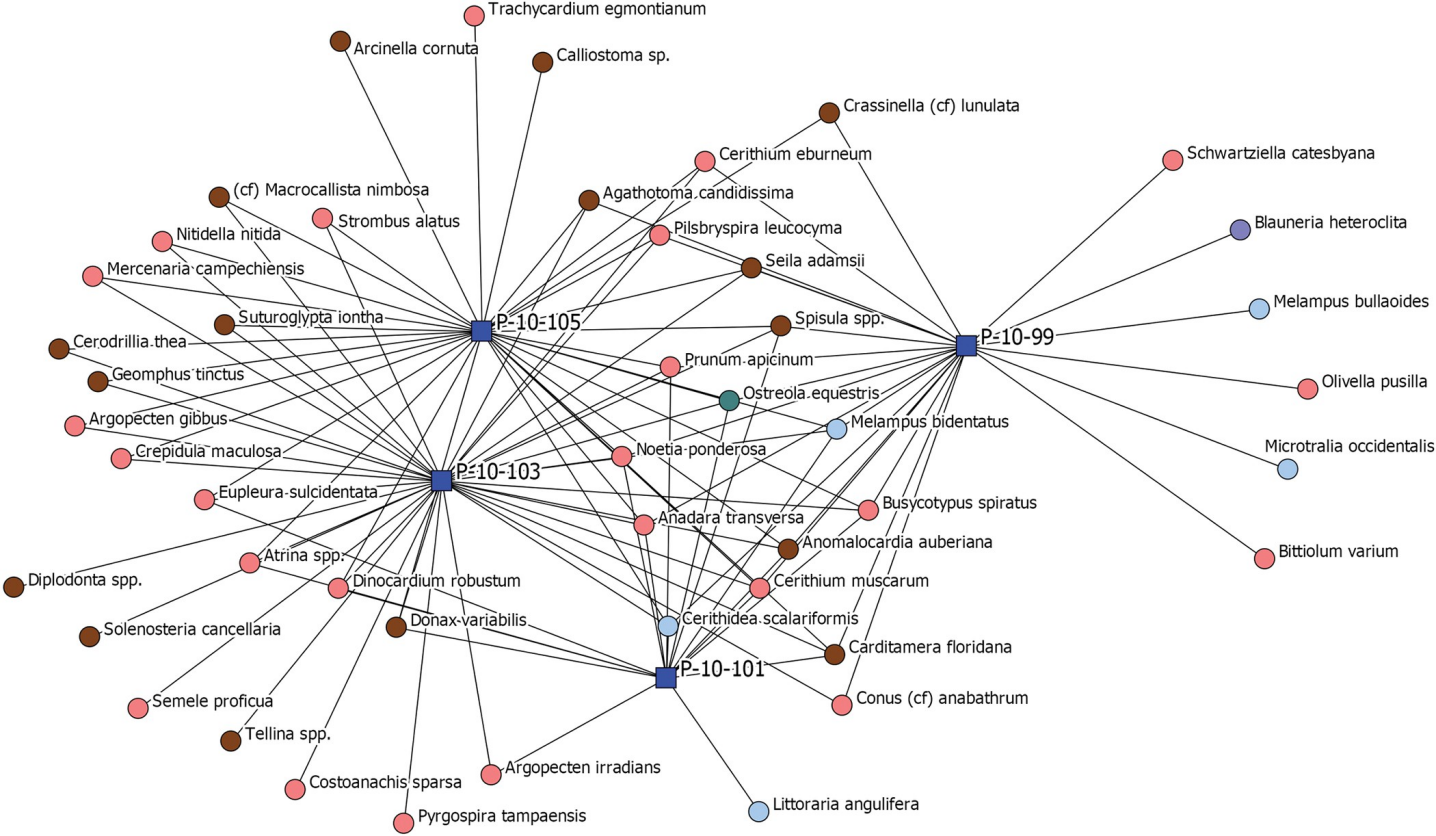
Two mode network depicting individual stratigraphic layers from Operation P (blue squares) and all invertebrate species identified in these layers that are found exclusively in a single kind of habitat (circles). The colors of the circles represent habitat types (seagrass meadows (pink); beachzone/littoral (brown); oysterbed community (green); mangrove swamp (blue); tidal stream (purple)). P-10-105 is the earliest deposits located at the bottom of the stratigraphic column and P-10-99 is the latest deposits located at the top of the column. Ties represent the presence of a species in a stratigraphic layer.

Fig 6 depicts a two-mode network with individual stratigraphic layers from Operation P (blue squares) and the habitat types of invertebrates that live exclusively in a single habitat found in each layer (red circles). P-10-105 is the earliest deposit located at the bottom of the stratigraphic column and P-10-99 is the latest deposit located at the top of the column. Ties represent the presence of a species in a stratigraphic layer.

**Fig 7:**
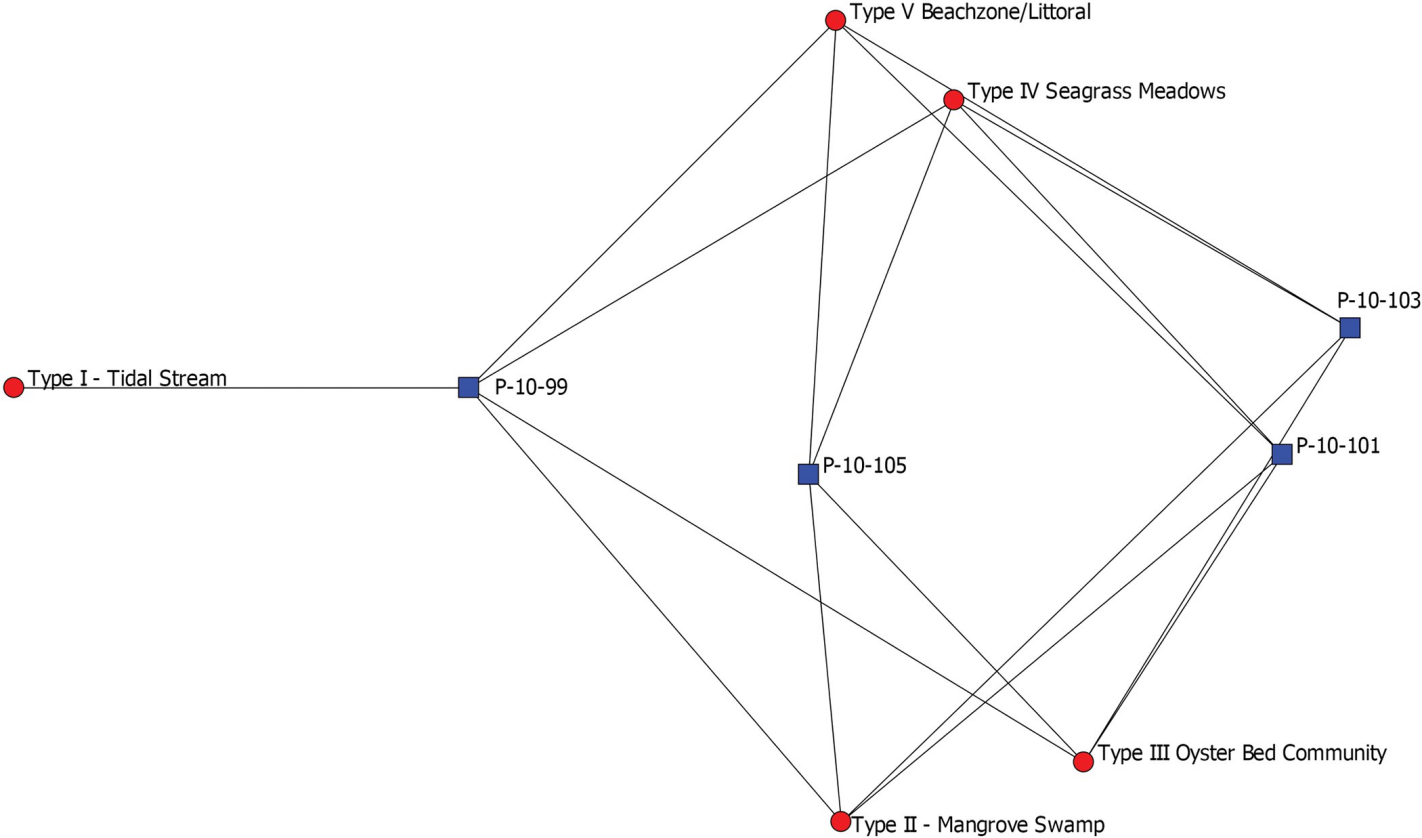
Two mode network depicting individual stratigraphic layers from Operation P (blue squares) and the habitat types of invertebrates found in each layer (red circles). P-10-105 is the earliest deposits located at the bottom of the stratigraphic column and P-10-99 is the latest deposits located at the top of the column. Ties represent the presence of a species belonging to a particular habitat in a stratigraphic layer.

Collapsing percent MNI of species into percent MNI of habitats represented, Fig 7 depicts stratigraphic layers (blue squares) and specific habitats (red circles). Levels 103 and 101 are similar in their compositions of habitats represented in their assemblages which include littoral zones, seagrass meadows, mangrove swamps, and oyster beds. Layer 105 is slightly different in terms of percent composition of these habitats. Layer 99 is even more dissimilar, characterized not only by a different composition of habitats, but also by the presence of species from tidal stream habitats. Overall, this represents a shift in species exploited through time primarily from higher salinity habitats to ones of lower salinity which can be linked both to shifting environmental conditions associated with the global onset of the Little Ice Age ca. AD 1250 as well as anthropogenic landscape modifications that likely altered freshwater runoff patterns, such as the construction of canals and water impoundment features.

**Fig 8:**
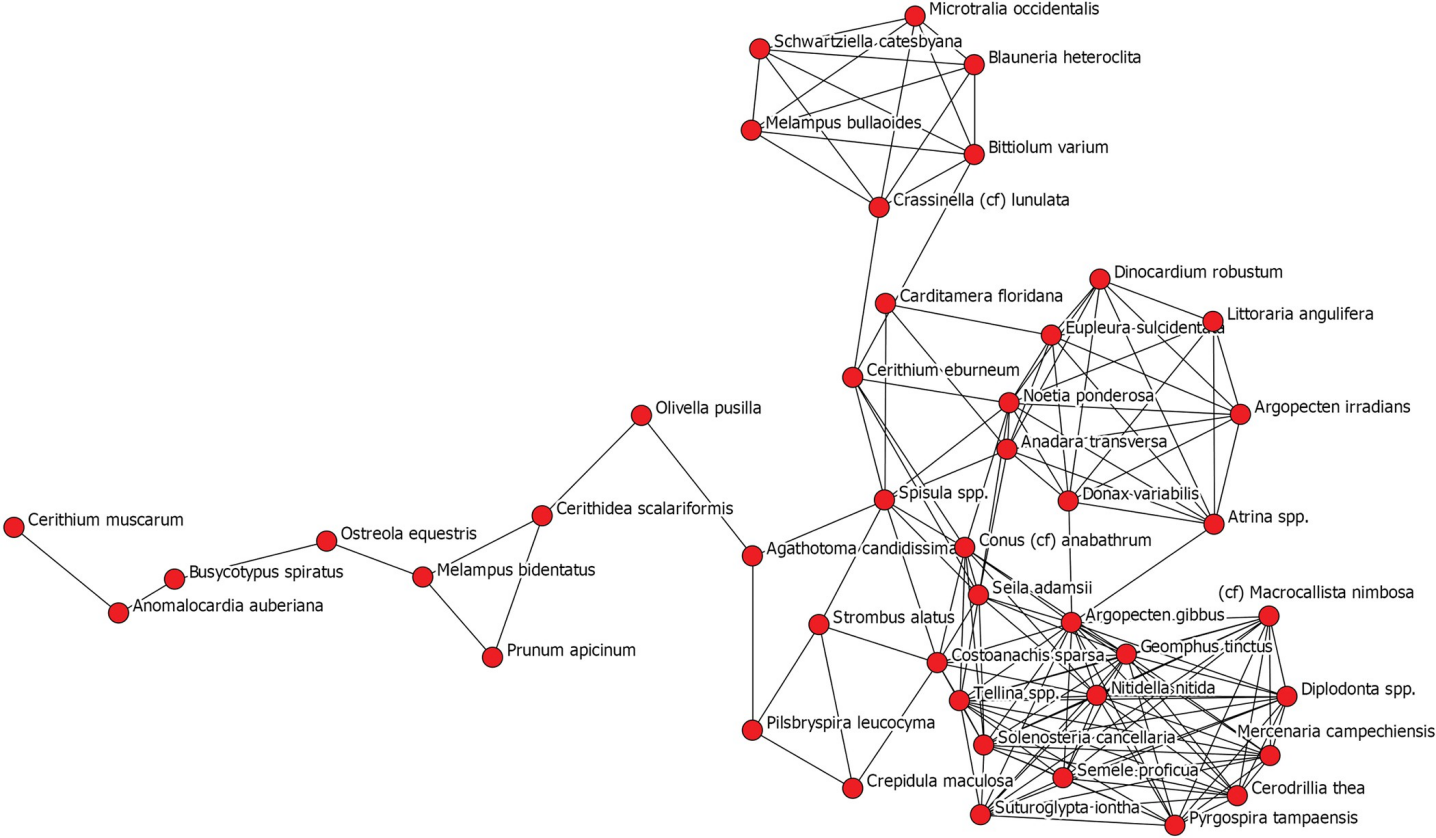
One mode network depicting all invertebrates that live exclusively within a single habitat type identified from all four analyzed layers of Operation P. Ties between species indicate similarity in presence and abundance across the four stratigraphic layers. Two species found in similar proportions across the same layers will have a tie linking them to one another. The closer two nodes are in the network, the more similar their abundance in the same contexts. A tie is present when the similarity between two species is greater than 60%.

**Fig 9:**
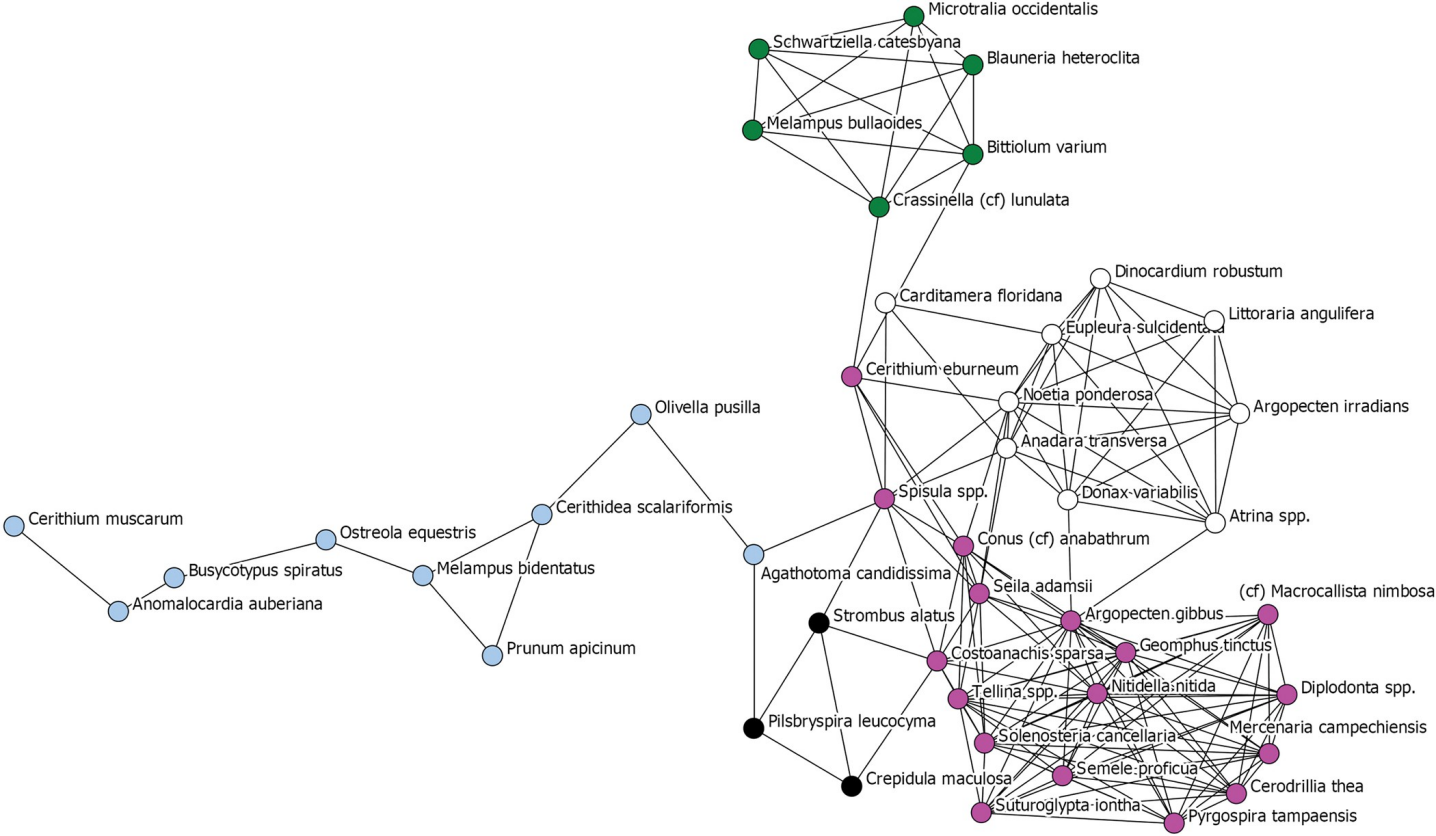
One mode network depicting all invertebrates that live exclusively within a single habitat type identified from all four analyzed layers of Operation P. Ties between species indicate similarity in presence and abundance across the four stratigraphic layers. Two species found in similar proportions across the same layers will have a tie linking them to one another. The closer two nodes are in the network, the more similar their abundance in the same contexts. Nodes are colored to reflect the results of the Girvan-Newman community-detection analysis. Each color represents a statistically significant sub-group within the overall network.

**Fig 10:**
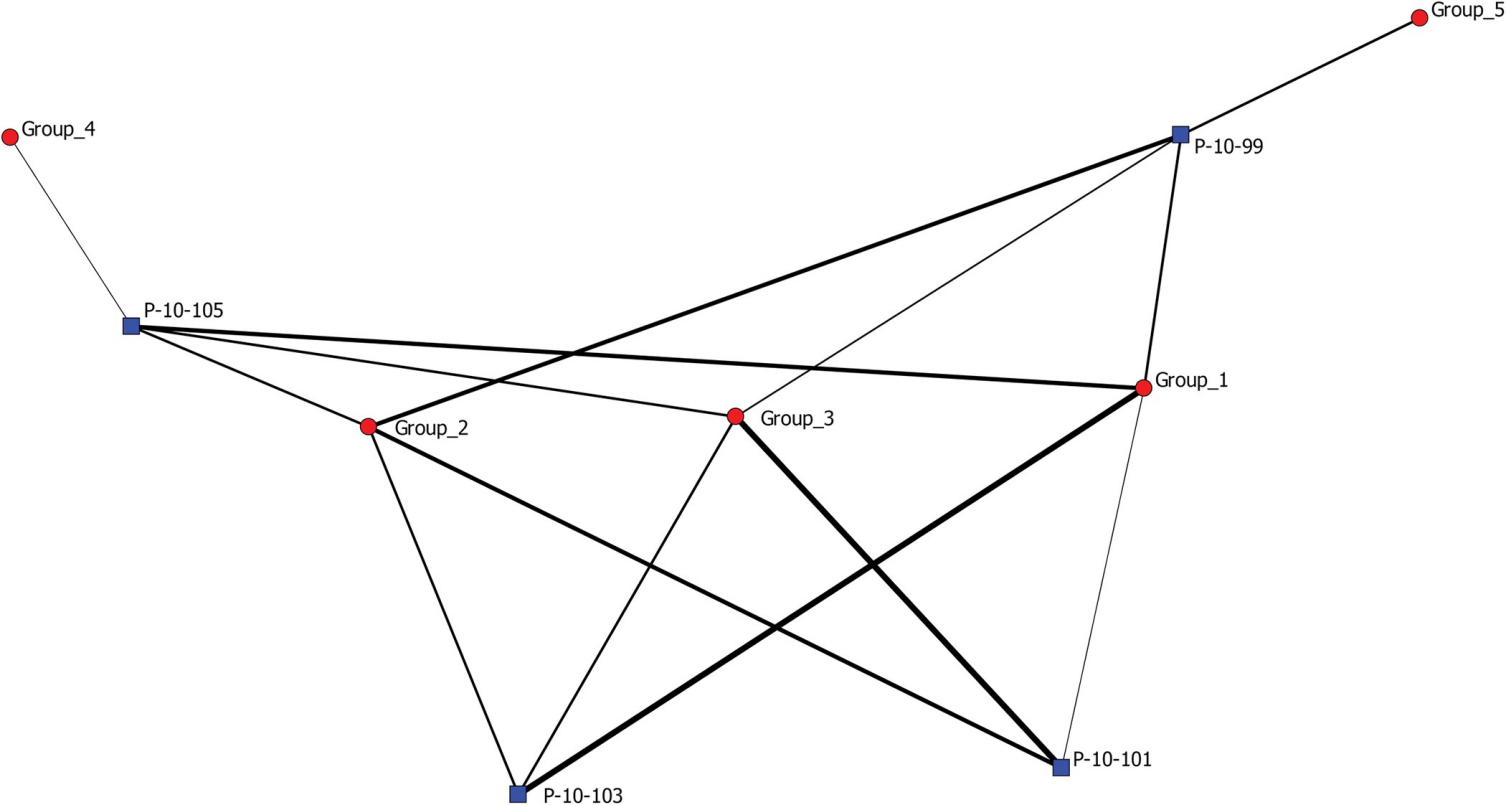
Two mode network depicting stratigraphic layers in Operation P (blue squares) and the sub-groups identified through the Girvan-Newman detection algorithm (red circles). These five sub-groups are depicted in Fig 9. A tie is present when a species belonging to a subgroup is present in a particular stratigraphic layer. The thickness of ties represents the abundance of species belonging to particular sub-groups in each stratigraphic layer.

Fig 8 depicts only species as nodes. A tie between two species indicates similar abundances across similar contexts, providing an immediate visual about which species are most commonly found together and in similar proportions. It also reveals potential sub-groupings. In this regard, we applied the Girvan-Newman method described above for community detection (Fig 9). Simply, these are statistically meaningful sub-groups within the network. Five subgroups are detected that represent groups of species that are most commonly found together in similar contexts. To quasi-test the utility of community-detection for interpreting our data, we circle back by assigning species to their identified sub-groups and then create a new two-mode network (Fig 10) depicting layers (blue squares) and algorithm-identified sub-groups (red circles). Ties are present between a sub-group and a context if species from a particular sub-group make up at least 5% MNI of the layer’s total assemblage and are weighted to reflect these percentages.

We note Group 3 species are found in all contexts and located at the center of the network. Group 5 species occur in substantive proportions in Layer 99 and unsurprisingly represent tidal stream species, lower salinity habitat, as depicted in a previous network. Layer 105 has the lone connection to Group 4 species. Layer 105 temporally occurs just prior to the onset of the Little Ice Age, during a proposed time of warmer, wetter conditions with potentially higher sea-levels, and higher local salinities. This group detection, at minimum, further supports the marked change in species composition of these deposits through time. While this quick analysis itself is circular, it demonstrates network graphs, and even certain network metrics, here community-detection algorithms, can be useful tools for highlighting meaningful patterns from data yielded from a single stratigraphic unit. Indeed, our application of network analyses to zooarchaeological data from a single stratigraphic column demonstrates the simple, yet robust utility of network visualizations in discerning meaningful ecological patterns from the archaeological record. In the site-wide analysis below, we explore more substantive analysis and interpretations, beyond simple visualization, that can be scaffolded by network analyses.

### Site-wide analysis

**Fig 11:**
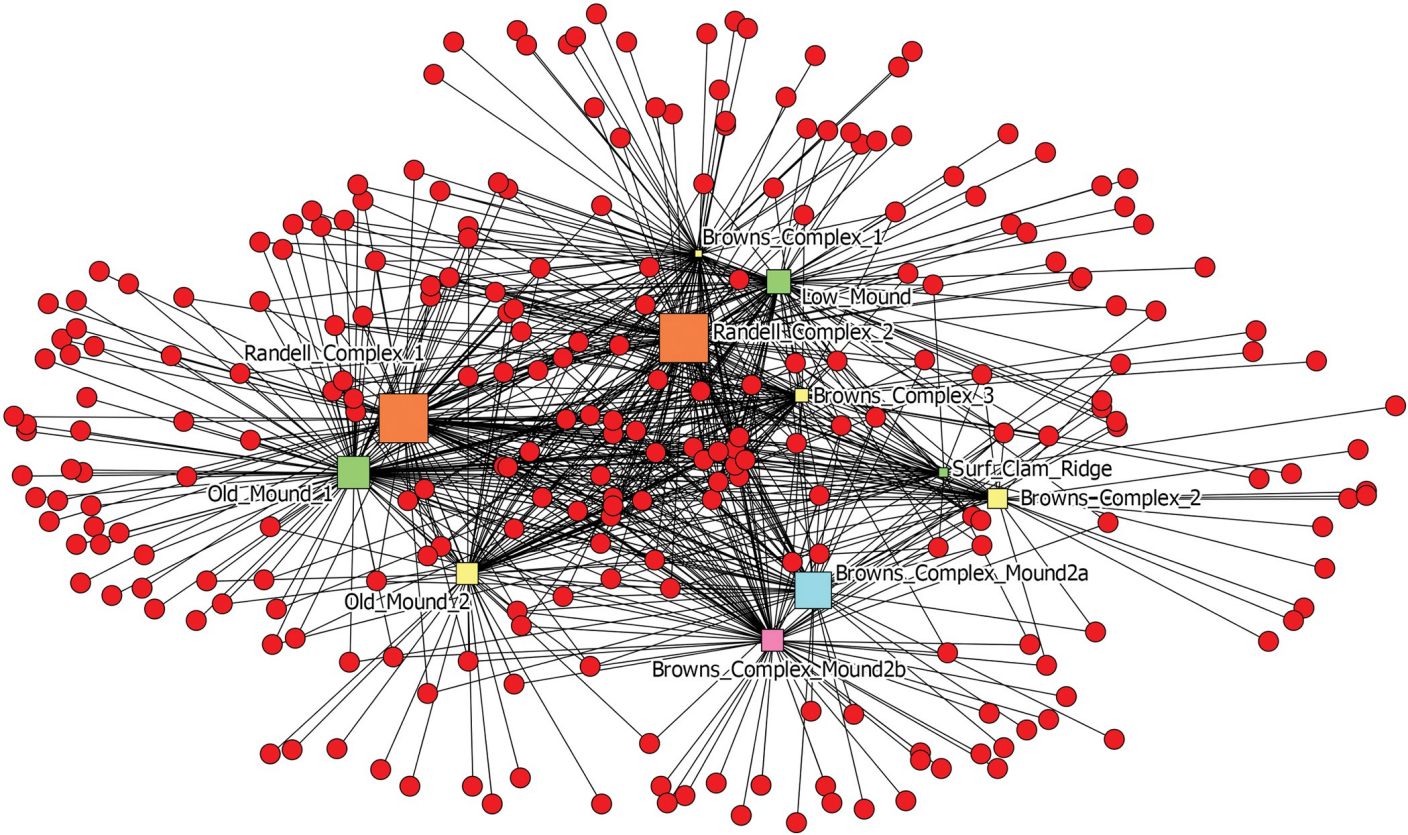
Two mode network depicting contexts from across Pineland (squares) and all species identified across these contexts (red circles). Contexts (squares) are sized by the diversity values of their zooarchaeological assemblages. Contexts are colored by time period (green—earliest through yellow, orange, and red—latest). A tie is present when a species is identified in a particular context.

**Fig 12:**
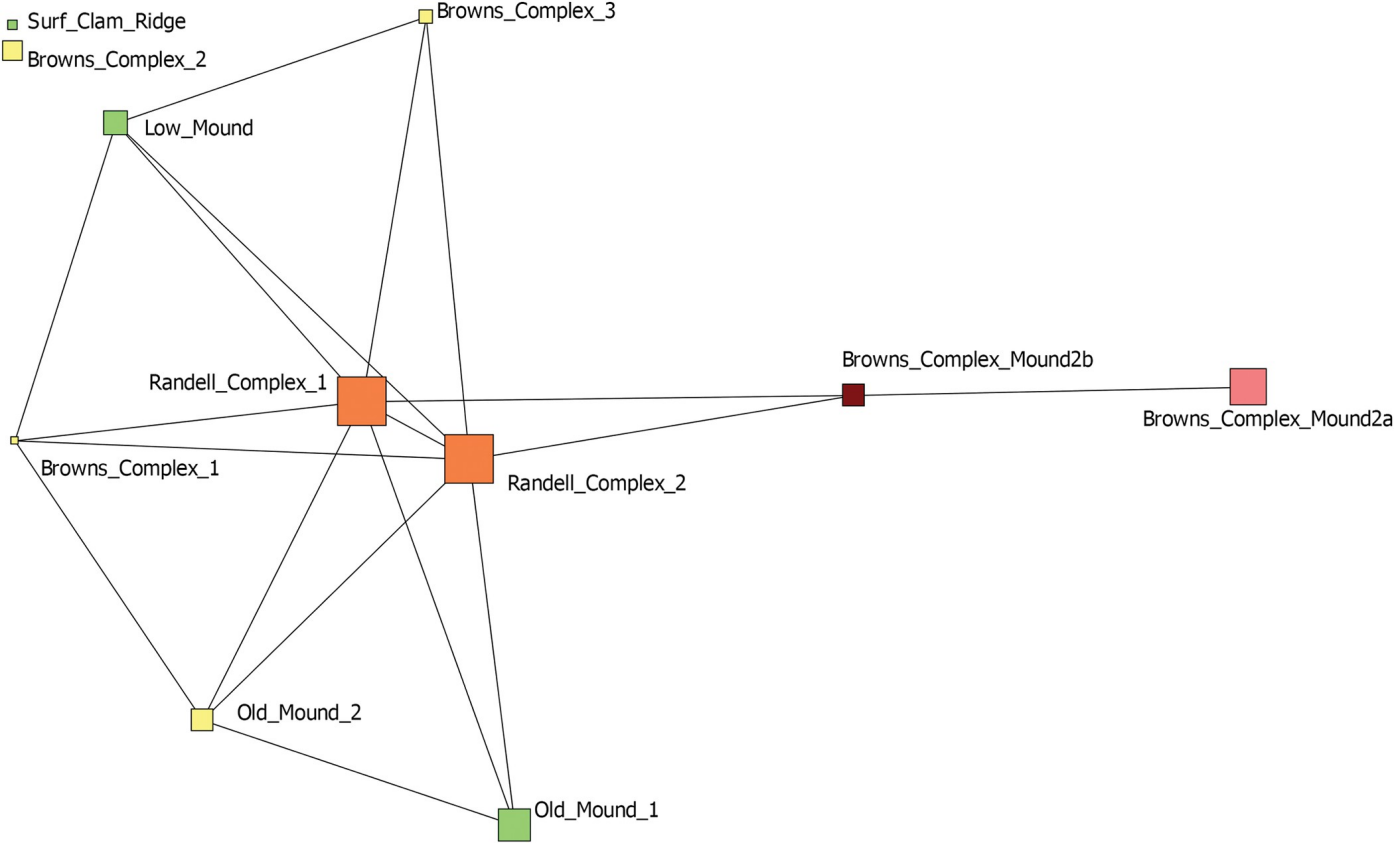
One more network depicting analyzed contexts from across the Pineland site. Nodes are sized to the diversity values of each context’s zooarchaeological assemblage. Contexts are colored by time period (green—earliest through yellow, orange, and red—latest). Ties represent similarities between context assemblages (e.g., kinds and quantities of species present). A tie is present when the similarity between two contexts have a Jaccard similarity coefficient of greater than 0.33.

Shifting to a site-wide analysis, Fig 11 depicts contexts from across the Pineland site (squares) and all of the species identified in these assemblages (red circles). Contexts are colored by time-period (earlier to later) and sized by assemblage diversity. To understand similarities and differences between assemblages, and thus variability in space and time, we once again use one-mode networks. This time, we calculated similarity scores using the Jaccard coefficient of similarity between contexts based solely on species presence/absence, not on percent MNI. The similarity values range from 0 (dissimilar) to 1 (similar). The chosen cutoff value for ties depicts the network graph just before it is broken apart, highlighting the dataset’s substantive structure and topology (Fig 12).

This one mode network is depicted as Fig 12 and includes only contexts, colored by time (green-earliest through yellow and orange to red-latest), and sized by assemblage diversity. Temporally, the earliest contexts, are on the left side of the graph, the slightly later contexts (orange) in the center, and the latest contexts (red) to the right. The presence/absence of particular species through time is clearly driving part of this network’s structure. Spatially, note two layers from Old Mound are located just next to each other at the bottom of the graph, Randell Complex assemblages located next to each other in the center of the graph, and Brown Complex Mound 2 assemblages located next to each other to the right. Indicating that space, and by extension social, political, or economic institutions, and not solely time or ecology, are driving the nature of the zooarchaeological record.

**Fig 13:**
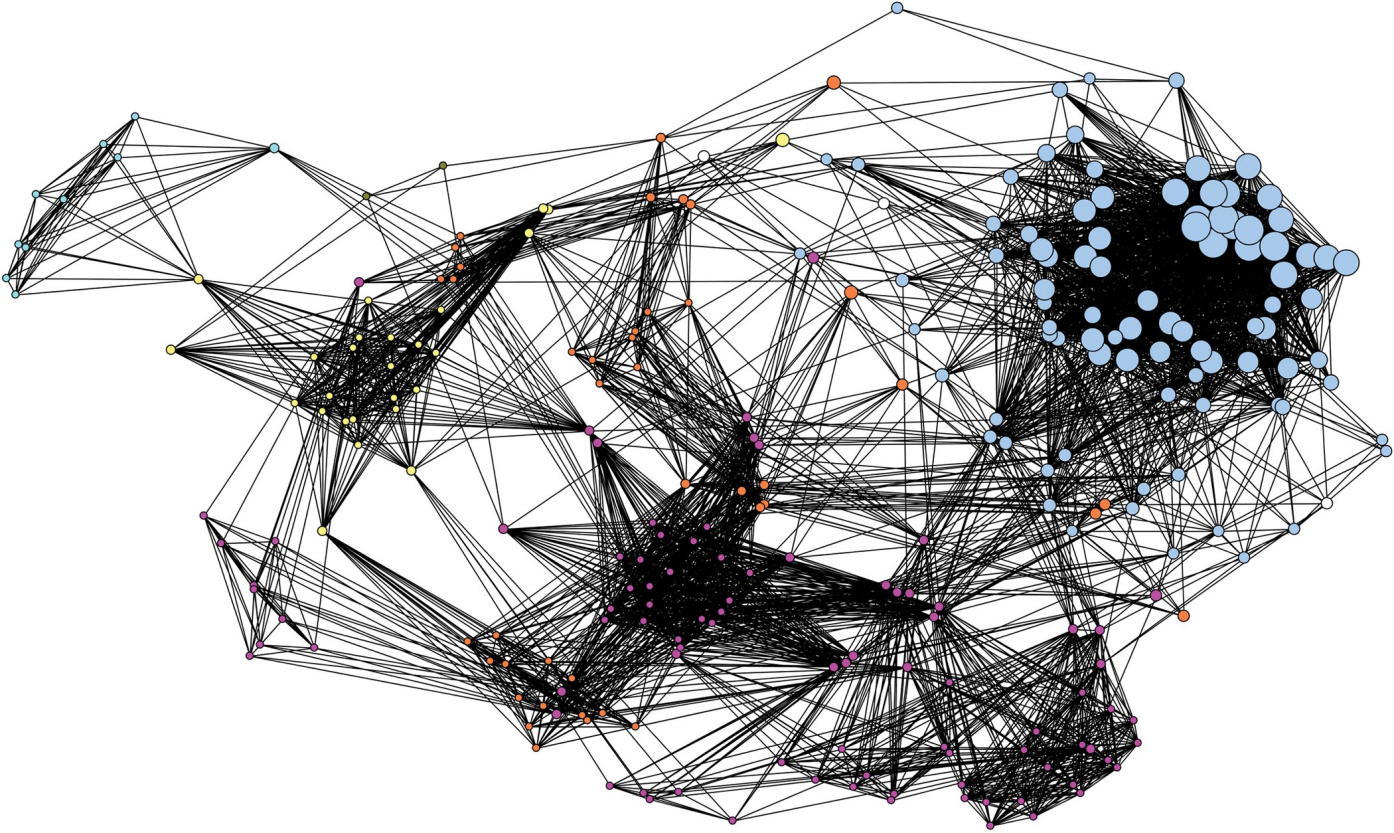
One mode network depicting all species identified across all contexts at the Pineland site. Ties between species indicate similarity in presence and abundance across Pineland site contexts. A tie is present when the Jaccard similarity coefficient for two species is greater than 0.40. Nodes are sized to reflect each species’ ubiquity value across contexts. Nodes are colored to reflect the results of the Girvan-Newman community-detection analysis. Each color represents a statistically significant sub-group within the overall network.

To further explore site-wide patterns, we once again created a one-mode network of only species (Fig 13). Here, species are nodes, colored by sub-group assignment as determined through another application of the Girvan-Newman community-detection algorithm, and sized by their ubiquity across Pineland contexts. The sub-group to the upper right of the graph, depicted as light blue, clearly represents a subgroup of species that share in their ubiquity values and thus in their presence across the majority of site contexts. The remaining subgroups are comprised of species with low ubiquity, highlighting how “packages” of species are likely tied to specific places and temporal periods across Pineland.

**Fig 14:**
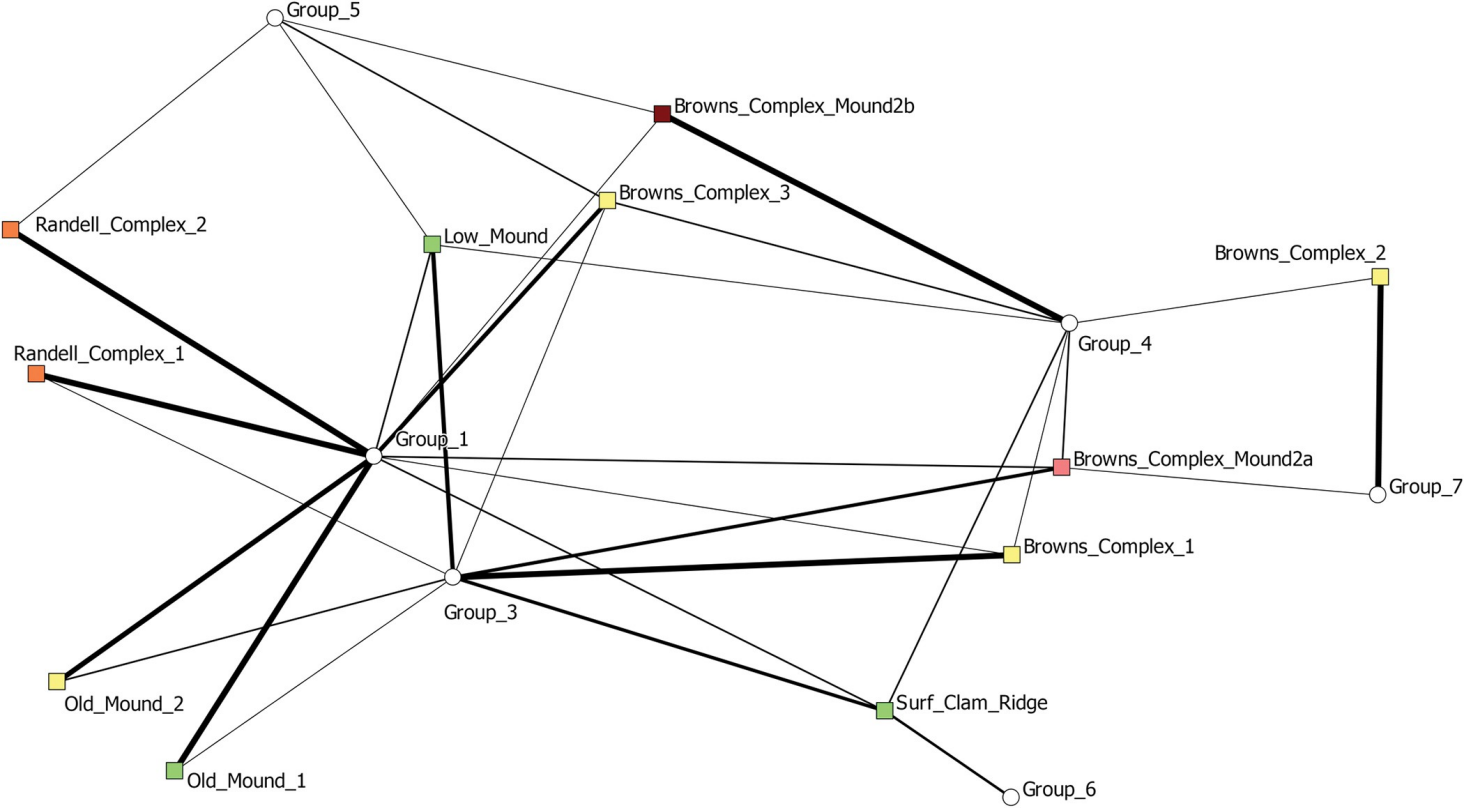
Two mode network depicting Pineland site contexts (squares) and the sub-groups identified through the Girvan-Newman detection algorithm (red circles). These five sub-groups are depicted in Fig 13. A tie is present when a species belonging to a subgroup is present in a particular context. The thickness of ties represents the abundance of species belonging to particular sub-groups in each context. Contexts are colored by time period (green—earliest through yellow, orange, and red—latest).

We then assigned species to their identified subgroups, this time there were seven, and visualized a two-mode network of contexts (squares) and sub-groups (white circles) (Fig 14). Similar to the one-mode network of contexts, we see a clear component of space driving the network structure. Brown’s Complex assemblages cluster to the right of the graph and are uniquely characterized primarily by species belonging to Groups 4 and 7. Old Mound contexts are grouped in the bottom left and are characterized by the presence of species from Groups 1 and 3. Randell Complex assemblages are to the left and characterized by species belonging to Groups 1 and 5.

**Fig 15:**
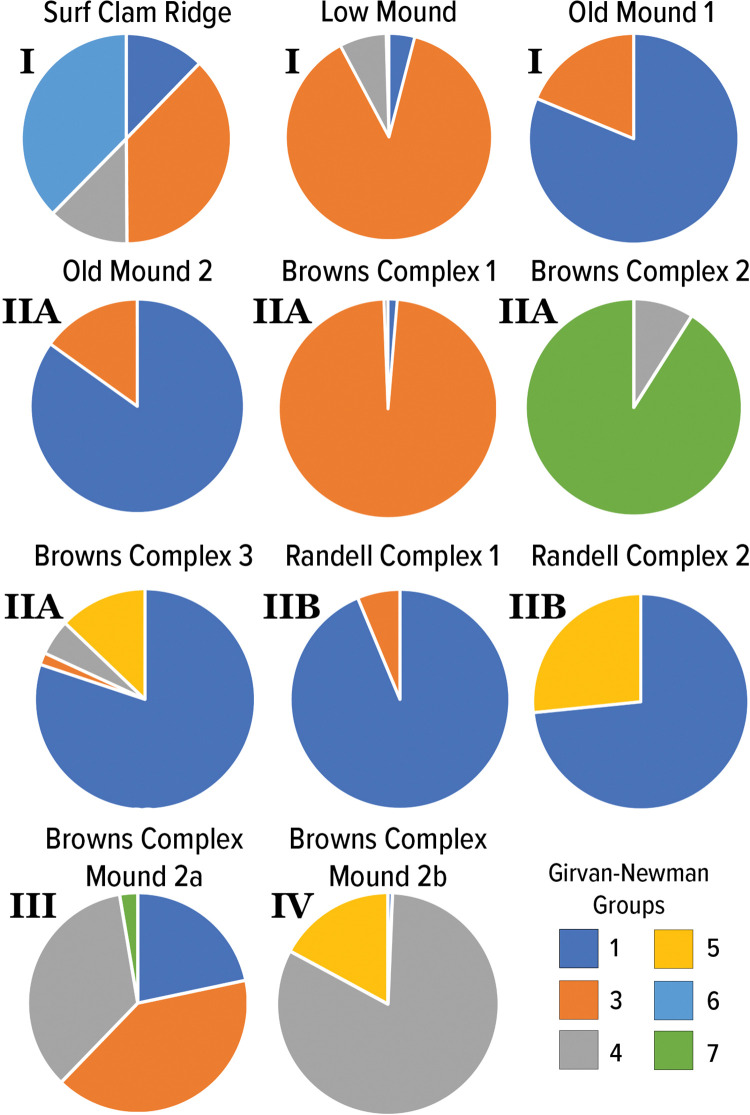
Pie charts depicting the proportions of each network-derived group present in each of the Pineland contexts. The optimal grouping solution was six groups. For these pie charts, we removed Group 2. As discussed below, Group 2 represented a package of species that was ubiquitous across the site and across contexts and likely represents a common “base” of species that were exploited. The other six groups are where we identifying and interpret substantive variability across contexts.

We actually find that one of the more significant utilities of network analysis is this group detection functionality. This ultimately provides a better tool for examining species cooccurrences in regard to how species are grouped together not only in space but also through time. With the presence of such large datasets, there can be a real difficulty in identifying patterns, especially when trying to make sense of species cooccurrences. Here, we dive deeper into the results of these group detection procedures and examine the changes in specific groups of species through time and space. As we demonstrate, important variation and patterning exists that is not captured through traditional approaches and analyses commonly employed in zooarchaeological research. Fig 15 illustrates that distribution of network-derived groupings across each of the Pineland contexts and serves as the basis of the following discussion.

Previously, deFrance and Walker [2] discussed differences in samples identified from strata across Pineland. These strata were differentiated based on contextual interpretations leading to two categories: general midden and house/activity areas. For these analyses the Old Mound 2, Brown’s Complex 2, and Brown’s Complex Mound 2a are categorized as house/activity areas while Brown’s Complex 1, Brown’s Complex 3, and Brown’s Complex Mound 2b are categorized as general midden strata. In this previous comparison, they argue that household/activity areas are marked by greater refuse from vertebrate taxa while general midden strata are dominated by food refuse from gastropods [2]. This trend is proposed to have changed over time, previous analyses identified a greater diversity of taxa in the Caloosahatchee IIA general midden samples, especially non-food taxa, when compared to those samples associated with household activity. In later time periods, Caloosahatchee III/IV, general midden deposits contain higher vertebrate diversity and overall abundance in terms of both taxa and MNI.

However, through the group detection analysis here, we demonstrate through network graphs that similarity in zooarchaeological assemblage is more driven overall by time than contextual, spatial, or intra-site differences in categorization but there is important contextual differentiation different that previously discussed. In fact, when compared against one another, the group detection results for similar typed contexts show markedly different, specific packages of species associated with each of these contexts. While previous arguments proposed that Caloosahatchee IIA general midden samples and those identified as house/activity areas demonstrate a greater degree of distinctiveness to one another than to the Caloosahatchee III/IV samples, our network analyses demonstrate that there is a significant level of intra-category diversity in taxa presence and abundance across contexts. For example, we find that Old Mound 2 (originally categorized as house/activity area) and Browns Complex 3 (originally classified as general midden) are both dominated by species assigned to Group 1 and diverge only in the abundance of Groups that make up a minority of each assemblage. There is clearly much greater complexity in deposit composition operating beyond the bounds of previous categorization of contexts.

The group detection results for Surf Clam ridge demonstrate the presence and distribution of groups not seen in other contexts with almost equal distribution of Groups 1, 3, 4, and 6. These groups capture the widest diversity of potential food resources represented in Pineland deposits. Surf Clam Ridge represents one of the earlier contexts analyzed, dating to the Caloosahatchee I period. Previous discussions of this sample center on the high abundance of surf clams (*Spissula solidissima*) in this deposit which, among other lines of evidence, led to interpretations of these deposits as post-storm deposits. The diversity of species packages represented here, however, demonstrate that the assemblage composition of these deposits may be much more complex than a storm-surge deposit might produce.

Similarly, analyses highlighted the association between pen shell (Pinnidae), sea urchin (Echinoidea), and surf clam as representative of storm deposits, since all three prefer marine habitats to the estuarine waters of Pine Island Sound, on the basis of their co-occurrence and on the basis that some were recovered still articulated [1]. However, in our group detection analyses, all three species were identified within the largest group, Group 2. We interpret the formulation of Group 2 as a “base” package of species. These species are almost ubiquitous across the site and are found commonly in high proportions with all other species in the group. None of these species, nor Group 2 as a whole, are unique to any particular type of context. As such, when the entire species package that is Groups 2 is examined holistically, the association between pen shell, sea urchin, and surf clams becomes negligible, highlighting the need to think critically about species co-occurrences and their interpretations in regards to past site formation processes. This is not to say these previous interpretations are incorrect but to again, highlight the complexity in interpreting species co-occurrences in the past, especially those recovered from anthropogenic deposits.

Interestingly, aside from Group 2’s ubiquity, there is little diversity of Groups at each context. That is, very specific packages of species can be used to define the assemblages of each context. Both samples from Old Mound are dominated by species present in Group 1 and a smaller percentage by Group 3. The species present in both Groups 1 and 3 are represented in almost equal proportions of those categorized as food and non-food taxa [2, 20]. The deposits at Old Mound are hypothesized as middens that accumulated under the water on the shoreline, attracting many non-food, estuarine invertebrate scavengers to the organic rich midden deposits resulting in higher percentages of non-food taxa.

Of particular note is the diversity in Group Detection Group Number in all three Brown’s Complex deposits meaning very specific species packages define each of these contexts. For example, Group 7 species dominate the Brown’s Complex 2 deposit, C-5-88-2 Stratum 8, dating to ca. 560–775 (95% probability) [1: Appendix A] which was deposited prior to mound construction. Based on estimated season of capture for both pigfish (*Orthopristis chrysoptera*) and pinfish (*Lagodon rhomboides*), this deposit accumulated in the fall, a time when the biomass available in both of these populations is at its highest [48]. The group that dominates this deposit, Group 7, contains ten separate taxon including 2 species of shark, (blacknose shark (*Carcharhinus* cf. *acrinotus*) and scalloped hammerhead (*Sphryna lewini*), the southern stingray (*Dasyatis americana*), barracuda (*Sphyraena* sp.), redear sardine (*Harengula humeralis*), killifishes (Cyprinodontidae), ladyfish (Elopidae), scallops (Pectinidae), rails (Rallidae), and mice (Cricetidae).

Barracuda, while commonly classified as an open marine fish, commonly occur among the nearshore seagrasses and mangroves, especially during their first two years of life, feeding on small schooling fish. Common prey include, among others, killifishes and herrings, also represented in this sample. Redear sardines are small nocturnal schooling fish commonly found in coastal waters and seagrass beds of southern Florida. Killifishes are generally very small, schooling fish with the ability to be highly adaptable in terms of environmental locale. Blacknose sharks are smaller bodied, nearshore sharks who commonly feed on small schooling fish. Scalloped hammerheads are a moderately sized shark who commonly feed on sardines and other schooling reef fish. All of these species identified as belonging to a single subgroup within the ecological network share important ecological relationships to one another. The predators of the group commonly feed upon the schooling fish in the group. While we do not have size/age class information for the barracuda or sharks, it is likely these specimens do not represent offshore fishing efforts but were likely bycatch associated with practices by humans that aimed to net their prey such as the killifishes and sardines. Alternatively, this could represent a diversity in fishing technologies with both net fishing targeting schooling sardines but also hook and line fishing used for sharks, ladyfish, barracuda, and stingrays. It is only with the identification of species cooccurrences in this way that we can begin to make important considerations and interpretations regarding not only important subsistence strategies but also important information for examining spatial relationships at the site-wide level. These contexts are directly mound-adjacent so thus are critical in how we situate processes such as anthropogenic landscape modification, differential access to resources, and so forth with the historical trajectories of social, economic, and political transformations.

The mound contexts for Brown’s Mound have the highest occurrence of species assigned to Group 4. Individually, many of these species within this group are highly mobile in regards to habitat and salinity preference. Only 5 of the 26 species assigned to Group 4 can be classified as non-food species. However, when evaluated collectively, Group 4 represents a package of species known to inhabit mangrove adjacent habitats. Of the non-food species, all prefer habitats such as oyster beds, tidal streams, and mangrove estuary environments. These two mound contexts from Brown’s Mound date to the two latest time periods, Caloosahatchee III (AD 1200–1350) and Caloosahatchee IV (AD 1350–1500), when we see the global climate shift into the Little Ice Age. Many of the fish assigned to Group 4 are relatively common fish within the estuary today such as sand seatrout (*Cynoscion arenarius*), spotted seatrout (*Cynoscion nebulosus*), black drum (*Pogonias cromis*), members of the grunt family (Haemulidae and *Haemulon* spp.), and members of the jack family (Carrangidae). Technologically, many of these fish are large and fast requiring hook-and-line technology, a shift away from the net-fishing technology that seems to have dominated the pre-mound contexts. The dominance of these Group 4 species in samples from later temporal periods begs for future inquiry into potential shifts in subsistence bases, shifts in uses of space, particularly mound vs. non-mound locales, shifts related to major changes in global climatic regimes, or the most likely, a combination of shifting forms of socio-ecological systems in the region as more forms of social organization and urbanization began to unfold.

## Conclusions and suggested applications

Here we have made the case for the use of network graphs as a powerful tool for the initial, exploratory analysis of zooarchaeological datasets. From each of these graphs we could produce numerous hypotheses about ecology, environment, and human behavior across different scales of both space and time. The network graphs themselves can serve as valuable tools for visualizing and communicating traditional zooarchaeological concepts as the analysis itself is based on standard datasets and data formats. We hope to have demonstrated at the very least how networks can be fruitfully employed to give conceptual form to past human-centered ecosystems and the complex engagements that produced these material reflections. Beyond our simple introduction highlighting how networks might supplement, complement, or reinforce traditional analyses, we propose that the use of these methods could actually contribute meaningfully to the expansion of traditional zooarchaeological approaches, analyses, and interpretation. We offer a few suggestions for future work and development in this regard.

The analyses presented here were situated at two different scales, that of a single stratified deposit and at the level of the entire site. Scaling up, network analyses have the potential to provide insight at the regional scale, too, where individual sites are represented as nodes in a network, tied together by site-wide assemblage similarity. While network analyses could be applied at any scale to explore the structure of zooarchaeological dataset like we have done here, they can, and in the future should, of course be used within robust theoretical frameworks. For instance, a site-wide or regional scale network might be used to assess critical differences or patterns in foodways, inequities in access to resources, or social, cultural, or political differences driving differences in exploited fauna. Just as archaeologists have explored social networks through ceramic data, food resources may certainly be evaluated within a network framework to understand differences and similarities in interactions, preferences, exploitation, and consumption of natural resources as food. For example, from a macro-regional perspective, a network spanning southern Florida, from the coast to the interior, may reveal meaningful sociocultural boundaries reflected in resource use and cuisine as encoded in the zooarchaeological record. Alternatively, such networks may reveal close relationships between foodways and ecological gradients as one moves from coastal through estuarine and freshwater wetlands.

Beyond species lists, we argue that network analyses could be used to explore more nuanced aspects of zooarchaeological datasets as well. For example, we might build a site-wide network where ties between contexts represent the presence or absence of particular elements. Which parts of fish are represented at particular contexts across a site? Or, at a regional scale, which deer elements are represented at which sites? These networks would thus serve to visualize similarities or differences in the activities that occur at specific places within particular spaces. Network graphs could be used to explore the movement of resources across spaces or the distribution of parts of an economic system, differentiating between, and highlighting the intensity of, places of production and consumption.

A key component of using network graphs to visualize data is the flexibility they afford. That is, any dataset that can be represented as a matrix can be evaluated using network analyses. As such, network analyses could provide an invaluable tool for formally integrating environmental datasets. For instance, in a matrix depicting contexts and the presence/absence of species in those contexts, both faunal and paleobotanical taxa could be included. Such analyses would yield the exact same kinds of networks depicted here, except nodes representing species would include both plant and animal species, potentially illuminating important associations between these different classes of resources. Indeed, the “packages” identified through community-detection algorithms would be composed of both plants and animals and could be used to expand our visualizations and analyses of human-centered ecosystems by yielding a more holistic representation. Even more simply, rather than adding new kinds of nodes, there are countless node attributes that could be integrated into the visualization of zooarchaeological data. In this paper, we variously visualized nodes by changing their colors based on habitat or time period, or changed their sizes by ubiquity or abundance measures. We could also imagine altering node symbology to reflect, for instance, nitrogen or oxygen isotope values. Further building key environmental information into the visualization and analyses of our datasets.

An exciting combination of isotopic and network analyses could be the formal analysis of, and deep time perspectives on, trophic levels and food webs. In the introduction we referenced key works and trends in Ecological Network Analysis. In fact, recent work by Smith et al. [57] is indeed leveraging the archaeological record as lens into the long-term evolution of, and human impacts on, food webs and trophic relationships. This is an excellent example of how archaeological data might be leveraged within a network framework to contribute beyond broader ecological investigations and challenges. We would argue that network analyses offer a tool for translating zooarchaeological data into meaningful terms that are comprehensible across ecological disciplines. In this way, we hope to have illustrated the value of network methods not only to archaeological and anthropological questions, but to critical interdisciplinary challenges that can only be met through interdisciplinary communication.
